# A Novel SCA3 Knock-in Mouse Model Mimics the Human SCA3 Disease Phenotype Including Neuropathological, Behavioral, and Transcriptional Abnormalities Especially in Oligodendrocytes

**DOI:** 10.1007/s12035-021-02610-8

**Published:** 2021-10-30

**Authors:** Eva Haas, Rana D. Incebacak, Thomas Hentrich, Chrisovalantou Huridou, Thorsten Schmidt, Nicolas Casadei, Yacine Maringer, Carola Bahl, Frank Zimmermann, James D. Mills, Eleonora Aronica, Olaf Riess, Julia M. Schulze-Hentrich, Jeannette Hübener-Schmid

**Affiliations:** 1 Institute of Medical Genetics and Applied Genomics, University of Tübingen, Tübingen, Germany; 2 Centre for Rare Diseases, University of Tübingen, Tübingen, Germany; 3 DFG NGS Competence Center Tübingen, Tübingen, Germany; 4 Interfaculty Biomedical Facility (IBF) Biotechnology lab, University of Heidelberg, Heidelberg, Germany; 5 Department of (Neuro)Pathology, Amsterdam UMC, University of Amsterdam, Amsterdam Neuroscience, Amsterdam, The Netherlands

**Keywords:** Spinocerebellar ataxia type 3, Machado-Joseph disease, Knock-in mouse model, Myelinating oligodendrocytes, Ataxin-3

## Abstract

Spinocerebellar ataxia type 3 is the most common autosomal dominant inherited ataxia worldwide, caused by a CAG repeat expansion in the *Ataxin-3* gene resulting in a polyglutamine (polyQ)-expansion in the corresponding protein. The disease is characterized by neuropathological, phenotypical, and specific transcriptional changes in affected brain regions. So far, there is no mouse model available representing all the different aspects of the disease, yet highly needed for a better understanding of the disease pathomechanisms. Here, we characterized a novel Ataxin-3 knock-in mouse model, expressing a heterozygous or homozygous expansion of 304 CAACAGs in the murine *Ataxin-3* locus using biochemical, behavioral, and transcriptomic approaches. We compared neuropathological, and behavioral features of the new knock-in model with the in SCA3 research mostly used YAC84Q mouse model. Further, we compared transcriptional changes found in cerebellar samples of the SCA3 knock-in mice and post-mortem human SCA3 patients. The novel knock-in mouse is characterized by the expression of a polyQ-expansion in the murine Ataxin-3 protein, leading to aggregate formation, especially in brain regions known to be vulnerable in SCA3 patients, and impairment of Purkinje cells. Along these neuropathological changes, the mice showed a reduction in body weight accompanied by gait and balance instability. Transcriptomic analysis of cerebellar tissue revealed age-dependent differential expression, enriched for genes attributed to myelinating oligodendrocytes. Comparing these changes with those found in cerebellar tissue of SCA3 patients, we discovered an overlap of differentially expressed genes pointing towards similar gene expression perturbances in several genes linked to myelin sheaths and myelinating oligodendrocytes.

**Supplementary Information:**

The online version contains supplementary material available at 10.1007/s12035-021-02610-8.

## Introduction

Spinocerebellar ataxia type 3 (SCA3) or Machado-Joseph disease (MJD) (OMIM: 109150) is the most common inherited ataxia worldwide. It is caused by a CAG repeat expansion in the *ATAXIN-3* (*ATXN3*) gene in exon 10, leading to an expanded polyglutamine (polyQ) stretch in the corresponding protein, the deubiquitinase ATXN3 [1, 2]. Therefore, it is one of nine so-called polyQ diseases (SCA1, 2, 3, 6, 7 and 17, Huntington disease (HD), spinobulbar muscular atrophy, and dentatorubral pallidoluysian atrophy) [3]. While healthy individuals present with 13-41 CAG repeats, SCA3 patients show an expansion of 55-86 CAGs in one allele of the *ATXN3* gene [4]. The resulting polyQ-expansion in the ATXN3 protein leads to ubiquitin-positive neuronal inclusions, also containing the disease protein ATXN3, demonstrating a typical hallmark of SCA3. While these inclusions may not be toxic [5, 6], misfolding and aggregation of the disease protein is proposed to be the central cause of the disease pathogenesis in polyQ diseases [7, 8]. The age-dependent intraneural accumulation and aggregation of the expanded protein is associated with neuronal dysfunction and cell loss, predominantly of the brainstem, cerebellum, and spinal cord [9, 10]. In most cases, the symptomatic phase of the disease occurs in the third or fourth decade of life and is characterized by motor abnormalities including gait ataxia, ocular symptoms, and cognitive disturbances later in life [4]. To date, few therapeutic approaches are available, but neurofilament light chain (NfL) and phosphorylated neurofilament heavy protein (pNfH) show great potential as biomarkers for neurodegenerative diseases in general, against which the progression of SCA3 in patients and in our knock-in (KI) model can also be well monitored [11].

Mouse models are important tools to investigate disease pathomechanisms, to discover treatment possibilities, or to study longitudinal disease progression and biomarker changes. While several SCA3 mouse models are available, the majority of these have the disadvantage of expressing cDNA under the control of artificial promoters, e.g. CMV, L7, rHtt, PrP or Purkinje cell (PC) specific promotors [12–17]. Further, a genetrap model, expressing only the N-terminal part of the murine Atxn3 protein, not containing a CAG repeat, presented with an ataxia-like phenotype [18]. Although these models enabled the investigation of selected SCA3 features, they have certain disadvantages like containing excessive numbers of transgene copies, unnatural expression patterns, incomplete protein expression, the lack of regulatory sequences, or even a combination of these drawbacks. In 2002, Cemal and colleagues presented the first full-gene model expressing the human *ATXN3* gene with all regulatory sequences, yet still expressing the endogenous mouse *Atxn3* gene [19]. In 2015, the first *Atxn3* KI models for SCA3 were introduced. These models hold the advantage of expressing a CAG expansion in the murine *Atxn3* locus under endogenous regulatory elements and at physiological levels. Ramani and colleagues presented a KI line containing 82 CAG repeats in the murine *Atxn3* locus [20, 21], while Switonski and colleagues generated a mouse in which the murine *Atxn3* gene was replaced by a humanized version with 91 CAG repeats [22]. Although these mice displayed aggregate formation and a mild phenotype, they did not reflect the complete human SCA3 phenotype. We know from animal models of HD, that it is not sufficient to introduce the same CAG expansion length in mice or rats that is found in the pathogenic human *huntingtin (HTT)* gene. Instead, several folds of this length are required, to trigger a phenotype [23]. Thus, we concluded that it is necessary to introduce a much longer CAG repeat in murine *Atxn3* than observed in human patients, to provoke a human-like phenotype.

Therefore, we introduce a novel SCA3 KI mouse model expressing a hyper-expansion of an interrupted repeat of 304 CAACAGs either in one or in both murine *Atxn3* alleles and compared several neuropathological and behavioral features to those found in the YAC84Q mice [19] and to a newly generated KI mouse line only expressing 97 CAACAGs. The interrupted repeat was introduced to establish a mouse line, which provides the advantage of a stable intergenerational expression. Howerver, it does not allow new insights into RNA toxicity or repeat associated non-ATG (RAN) translation. Nevertheless, our new line is characterized by a massive formation of ubiquitin (Ub)-positive Atxn3 aggregates in brain regions vulnerable in SCA3 patients, accompanied by a mild loss of PCs. Further, mice showed a reduction in body weight, size, and additional physical changes accompanied by a motor phenotype including gait and balance instabilities as well as footprint abnormalities comparable to human SCA3 clinical features. Moreover, cerebellar transcriptome analysis revealed an age-dependent increase in differentially expressed genes (DEGs) clustering in distinct gene expression patterns. When comparing these changes in mice to post-mortem cerebellar data from SCA3 patients, an intriguing overlap of DEGs associated with myelinating oligodendrocytes became apparent, indicating shared perturbations between both organisms. Another feature that makes our SCA3 KI mice a suitable model is the fact that, like SCA3 patients as well as patients suffering from other neurodegenerative diseases, they secrete the protein NfL into the blood, which is recognized as a biomarker for neuronal loss in neurodegenerative processes [11]. Thus, our SCA3 KI mice represent an ideal model to study novel therapeutic approaches such as micro RNA (miRNA), as previously shown by Martier et al. [24], as they represent both the novel clinical and therapeutic biomarkers and map the human neurological phenotype.

Taken all these findings together, we present a new SCA3 KI mouse model mimicking the human patient SCA3 phenotype on a behavioral, neuropathological, and transcriptomic level, enabling the analysis of early onset events, longitudinal developments, as well as treatment and biomarker progression like NfL.

## Material and Methods

### Ethical Use of Animals

All mice were maintained by animal care staff and veterinarians of the University of Tübingen. All procedures were performed according to the German Animal Welfare Act and the guidelines of the Federation of European Laboratory Animal Science Associations, based on European Union legislation (Directive 2010/63/EU). Animal experiments were approved by the local ethics committee (Regierungspräsidium Tübingen (HG3/13)).

### Ethical Use of Human Tissue

All the work involving human tissue has been carried out in accordance with the Code of Ethics of the World Medical Association (Declaration of Helsinki) and with national legislation as well as our institutional guidelines.

### Generation of KI Lines

The KI founder lines were generated by Zink-finger technology (Zn-finger) as described previously [25]. The DNA binding domains of the Zn-finger nucleases were located 3’ and 5’ of the CAA(CAG)_5_ region (binding sequence: 5’-acagatattcacgtttgaatgtttcaggCAACAGCAGCAGCAGCAG-gaggtagaccgacctggacccctttcat-3’) in the murine *Atxn3* gene. The associated restriction site generated a double–strand break (DSB) within the sequence. A donor vector with (CAACAGCAG)_48_ interrupted repeats, flanked by 800 bp up- and downstream of the C57Bl/6 CAA(CAG)5 region was used to insert the specific mutation by homologous recombination (HR). In this process we generated two different SCA3 KI founder lines, the 304Q, which was further bred heterozygous and homozygous and is the focus in this study. We also generated the 97Q line, whose properties are shown in comparison with the 304Q lines, since it is comparable to the lines of Ramani et al. [20] and Switonski et al.[22].

### Genotyping by PCR

DNA was extracted from mouse ear biopsies using High Pure PCR Template Preparation Kit (Roche, Mannheim, Germany) according to the manufacturer’s instructions. Extracted DNA was used for genotyping by polymerase chain reaction (PCR) (Taq DNA polymerase, Qiagen, Hilden, Germany), using primers flanking the endogenous CAG repeat in exon 10 of the murine *Atxn3* gene (primer sequences, Table 1) and the following PCR conditions: 1 cycle 95 °C/5 min; 35 cycles of 95 °C/30 sec, 55 °C/30 sec, 72 °C/60 sec; 1 cycle 72 °C/10 min. The genotyping of the hemizygous YAC84Q transgenic mice for behavioral and neuropathological comparison in this study were performed by using primers only recognizing the human *ATXN3* cDNA (primer sequences, Table 1) and the following PCR conditions: 1 cycle 95 °C/5 min; 35 cycles of 95 °C/30 sec, 55 °C/30 sec, 72 °C/60 sec; 1 cycle 72 °C/10 min. PCR products were separated on a 1% agarose gel and visualized using a 1% ethidium bromide solution (Carl Roth, Karlsruhe, Germany).

**Table 1:** Primer sequences used for genotyping and qRT-PCRs

Gene	Species	Direction	sequence 5'-3'	Application
*Ataxin-3*	mouse	forward	TCA GGC AGT GAC CAT TTT GA	Genotyping/Fragment length KI mice
		reverse	CCT GAA CTT GTG GTC GGT CT	
*Ataxin-3*	mouse	forward	GAA TAT TTT AGC CCT GTG GAA TT	Genotyping YAC84Q mice
		reverse	AGT TTA AAA TCA GTA CCT GTA AAA ACG	
*Ataxin-3*	mouse	forward	CCA GTG ACT ACT TTG ATT CG	Fragment length YAC84Q mice
		reverse	TGG CCT TTC ACA TGG ATG TGA A	
*Ataxin-3*	mouse	forward	AGG CAA GCA GTG GTT TAA CT	qRT-PCR
		reverse	TGG CAG ATC ACC CTT AAC AAC A	
*Igfbp5*	mouse	forward	CAA CGA AAA GAG CTA CGG CG	qRT-PCR
		reverse	ACC TTG GGG GAG TAG GTC TC	
*Il20rb*	mouse	forward	CAG ACA CCT TGA AAA TAA CCA G	qRT-PCR
		reverse	CAG ACC CCA AAG AGA TGC TC	
*Il33*	mouse	forward	TCC AAC TCC AAG ATT TCC CCG	qRT-PCR
		reverse	CAT GCA GTA GAC ATG GCA GAA	
*mActb*	mouse	forward	CCA CAC CCG CCA CCA GTT CG	qRT-PCR
		reverse	TAC AGC CCG GGG AGC ATC GT	
*Mbp*	mouse	forward	TAC CTG GCC ACA GCA AGT AC	qRT-PCR
		reverse	GTC ACA ATG TTC TTG AAG	
*Mobp*	mouse	forward	GAG GAG GAC TGG ATC TGC TG	qRT-PCR
		reverse	TCA CTT CTT CCT TGG GGT TG	
*Pdh*	mouse	forward	GTA GAG GAC ACG GGC AAG AT	qRT-PCR
		reverse	TGA AAA CGC CTC TTC AGC A	
*Plp1*	mouse	forward	GTA TAG GCA GTC TCT GCG CTG AT	qRT-PCR
		reverse	AAGTGGCAGCAATCATGAAGG	
*Sdha*	mouse	forward	GCA GCA CAG GGA GGT ATC A	qRT-PCR
		reverse	CTC AAC CAC AGA GGC AGG A	
*Syndig1l*	mouse	forward	CAG TCA GGA GGA GGA GAG CG	qRT-PCR
		reverse	GCA GCA ATG CCC AGA GGC C	
*Tbp*	mouse	forward	TCT ATT TTG GAA GAG CAA CAA AGA C	qRT-PCR
		reverse	GAG GCT GCT GCA GTT GCT A	

The CAG repeat size in YAC84Q mice were analyzed as described in Cemal and colleagues [19]. Shortly, two primers amplifying the CAG repeat motif were used (Table 1). Reverse primer was labeled with Cy5-fluorophore and repeat size was analyzed and calculated using fragment analyzer (Beckman Coulter, Brea, USA). Only YAC84Q mice with a CAG repeat size from 80 to 85Qs were integrated in the respective analyses. Similarly, intergenerational stability of 97Q and 340Q CAA/CAG knockin mice were determined by fragment analyses (Beckman Coulter, Brea, USA or Agilent Technologies, Santa Clara, USA, respectively) using the following forward 5´-tcaggcagtgaccattttga-3´ and Cy5-labbeled reverse primer 5´-cctgaacttgtggtcggtct-3´. For both, PCR amplification was performed using Qiagen Taq polymerase, 2 mM dNTPs, 10mM from each primer and 100 ng DNA.

### Sequencing of CAG Expansion by Sanger Sequencing and Determination of CAG Length by Fragment Length Analysis

To analyze the right order of the CAACAGCAG expansion Sanger Sequencing was performed. After amplifying the CAG region by PCR (genotyping conditions), the PCR product was purified using the Qiaquick PCR Purification Kit (Qiagen, Hilden, Germany) according to the manufacturer’s instructions. Sequencing was performed with the BigDye Terminator v3.1 Cycle Sequencing Kit (Applied Biosystems, Waltham, USA) using 15-30 ng purified PCR product and the following PCR conditions: 1 cycle 96° C/2 min; 30 cycles of 96° C/10 sec, 55° C/5 sec, 60° C/3 min; 1 cycle 60° C/10 min. Sequencing was performed with either forward or reverse primer, separately. Sequencing products were cleaned up using magnetic beads (CDTR-0050; CleanNA, Waddinxveen, The Netherlands) and according to the Agencourt AMpure PCR purification (Beckman Coulter, Brea, USA) protocol. Separation of the sequences was performed on the ABI 3730xl DNA Analyzer (injection time: 5 sec, run duration: 40 min, separation gel: POP7, Applied Biosystems, Waltham, USA).

Capillary electrophoresis was used to determine the fragment length of the PCR products. A total of 25 μl of the PCR product was cleaned using 1X Agencourt AMPure XP beads (Beckman Coulter, Brea, USA) and eluted in 25 μl TE buffer. Concentration was measured using Qubit dsDNA HS (Cat No Q32854, ThermoFisher Scientific, Waltham, USA). Fragment size was determined by loading 1 ng/μl cleaned up PCR product on the Fragment Analyzer System (Agilent Technologies, Santa Clara, USA) using the qualitative DNA Kit dsDNA Reagent 35-5000bp (Cat No. DNF- 915, Agilent Technologies, Santa Clara, USA).

### Animal Housing and General Health Assessment

All animals were housed under standard conditions in type II long cages. After weaning, they were kept with a maximum of five animals of the same sex per cage without enrichment, avoiding animals housing alone. They were maintained within a 12-hour light-dark cycle and had access to food and water ad libitum. Measurement of body weight and a thorough inspection of the health status of the animals were performed every alternate week.

### Accelerating RotaRod Test

Twelve to fifteen animals per genotype (WT/WT or KI mice WT/304Q and 304Q/304Q; males *n* = 7–8; females *n* = 6–7) were placed on an accelerating RotaRod (TSE-Systems, Bad Homburg, Germany) on four consecutive days. Day one to three were training sessions, each with an acceleration from 4 to 16 rpm in 2 min. Two rounds of training were performed per training session. Day four was the test session with two test trials, each test trial with an acceleration from 4 to 40 rpm in 5 min. The time the mice spend on the accelerating rod was recorded. Each training and test session started at the same time of the day and two trials per day were performed. Mice could rest for 1 hour between trials. Accelerating RotaRod test was performed longitudinal at 3, 6, 9, 12, 15, and 18 months of age. For comparison of the KI mice with the YAC84Q transgenic mice a second set of animals, comprised of 15 wildtype (WT/WT*) (male *n*= 6, female *n* = 6–9) and 16 YAC84Q (male *n* = 10, female *n* = 4–6) mice were analyzed separately from the KI cohort (* = control group to YAC84Q mice).

### Gait Analysis

Gait analysis was performed with the gait analysis system Catwalk 8.1 (Noldus, Wageningen, The Netherlands). Twelve to fifteen animals per genotype (males *n* = 7–8; females *n* = 6–7) were placed individually in a tunnel on a glass plate where the mice could move voluntarily. Each mouse had to perform five runs with minimal run duration of 0.5 sec and a maximum of 10 sec. The maximum variation in speed was 60%. Runs with rearing behavior or a change of direction were automatically discarded. Evaluations of the runs were performed with the Catwalk X software (Noldus, Wageningen, The Netherlands). Gait analysis was assessed at 6, 12, and 18 months of age. For comparison of the KI mice with the hemizygous YAC84Q transgenic mice a second set of animals, comprised of 15 wildtype (WT/WT*) (male *n*= 6, female *n* = 6–9) and 16 YAC84Q (male *n* = 10, female *n* = 4–6) mice were analyzed separately from the KI cohort with 12 and 15 months of age.

### Tissue Preparation for RNA and Protein Analyses

To investigate protein and RNA levels, YAC84Q, WT/97Q (only for protein studies), WT/304Q and 304Q/304Q KI mice and their WT/WT littermates were sacrificed by CO_2_ inhalation followed by head decapitation (*n* = 3, per genotype and experimental setup). Brain regions and peripheral organs for protein analysis were immediately dissected and snap-frozen in liquid nitrogen. Cerebellar samples for RNA analysis were stabilized overnight in RNAlater (Qiagen, Hilden, Germany). All samples were stored at -80 °C until further usage.

### Tissue Homogenate and Lysate for Protein Analyses

For tissue lysate and homogenate preparation of mouse samples, frozen tissue was mechanically homogenized in TES buffer (4% Tris Base pH 7.5, 0.1 mM EDTA, 100 mM Na2Cl) containing protease inhibitor cOmplete, EDTA-free Protease Inhibitor Cocktail (Roche, Mannheim, Germany) with the VDI 12 homogenisator (VWR, Darmstadt, Germany). TNES (TES-buffer + 10% Igepal CA-630) was added in a relation of 1:10 and incubated on ice for 30 min. Part of the homogenates was stored at – 80° C and later used for filter retardation assay. The rest of the homogenates were centrifuged at 13.200×*g* for 25 min at 4 °C and the supernatants (lysates) were transferred to a new tube. Glycerol was added to a final concentration of 10%. Lysates of human cerebellar tissue were obtained in the same way, but RIPA buffer (50 mM Tris pH 7.5, 150 mM NaCl, 0.1% SDS, 0.5% sodium deoxycholate and 1% Triton X-100) was used instead of TES [26]. RNA samples from the same patients were later included in the RNA-seq analysis of the human patients. Protein concentration was measured spectrophotometrically by Bradford Assay (Bio-Rad Laboratories, Feldkirchen, Germany).

### Western Blot Analysis

Western blotting was performed using standard procedures. In short, 4× LDS sample buffer (1 M Tris Base pH 8.5, 2 mM EDTA, 8% LDS, 40% glycerol, 0.025% phenol red) and 100 mM 1,4-dithiothreitol (DTT) (MerckMillipore, Darmstadt, Germany) was added to 30 μg of protein lysates and heat-denatured at 70 °C for 10 min. Adjacent, protein samples were separated by electrophoresis using a 8% Bis-Tris gel and MOPS (50 mM MOPS, 50 mM Tris Base pH 7.3, 3.5 mM SDS, 1mM EDTA) or MES (50 mM MES, 50 mM Tris Base pH 7.3, 0.1% SDS, 1 mM EDTA) electrophoresis buffer. Proteins were transferred on Amersham Protran Premium 0.2 μm nitrocellulose membranes (GE Healthcare, Solingen, Germany) using a Bicine/Bis-Tris transfer buffer (25 mM Bicine, 25 mM Bis-Tris pH 7.2, 1 mM EDTA, 15% methanol) and a TE22 Transfer Tank (Serva, Heidelberg, Germany) at 80 V for 2 hours by 4 °C. Afterward, membranes were blocked in 5% skimmed milk powder (Sigma-Aldrich, Munich, Germany) in TBS (Tris-buffered saline) (1 M Tris, 5 M NaCl) for 1 hour at room temperature and incubated with primary antibodies (mouse anti-α-tubulin, 1:5000, CP06, MerckMillipore, Darmstadt, Germany; mouse anti-Ataxin-3, 1:2500, clone 1H9, MAB5360, MerckMillipore, Darmstadt, Germany; rabbit anti-Ataxin-3, 1:500, clone 13H9L9, ThermoFisher Scientific, Waltham, USA; mouse anti-β-actin, 1:5000, A5441, Sigma-Aldrich, Munich, Germany ; mouse anti-GAPDH, clone GA1R, ab125247, Abcam, Cambridge, UK; rabbit-IL20rb; 1:700, LS-C804694, LSBio, Seattle, USA; rabbit anti-IL-33, 1:400, LS-C294906, LSBio, Seattle, USA; mouse anti-Mbp, 1:100, sc-66064, Santa Cruz, Dallas, USA; rabbit anti-Olig2, 1:2500, AB9610, MerckMillipore, Darmstadt, Germany; rabbit anti-Syndig1l, 1:400 SAB1301276, Sigma-Aldrich, Munich, Germany; rabbit anti-Vinculin, 1:1000, 13901, CellSignaling, Danvers, USA) overnight at 4 °C. Subsequently, membranes were washed with TBST (TBS + 0.1% Tween 20) and incubated with secondary antibodies (peroxidase affinePure donkey anti-mouse IgG H+L, 1:12000, 715-035-150, Jackson ImmunoResearch, Ely, UK; IRdye 800CW goat anti-mouse IgG (H+L) 1:1000, 926-32210, LI-COR, Bad Homburg, Germany; IRdye 800CW goat anti-rabbit IgG (H+L) 1:1000, 926-32211, LI-COR, Bad Homburg, Germany; goat anti-rabbit IgG H&L (HRP), 1:10.000, ab97051, Abcam, Cambridge, UK) for 1.5 hours at room temperature. In case of chemiluminescent detection, WesternBright Sirius Western blotting detection kit (Biozym, Hessisch Oldendorf, Germany) was used in the ratio 1:1:1 with one part TBS. Fluorescence or chemiluminescent signal detection was performed on the LI-COR ODYSSEY FC and quantified using the ODYSSEY Server software version 4.1 (LI-COR, Bad Homburg, Germany).

### Filter Retardation Assay

Filter retardation assay was used to detect SDS-insoluble Atxn3 and Ub species in brain tissue homogenates. Therefore, 12.5 μg of total protein was diluted in Dulbecco’s phosphate-buffered saline (DPBS) (Gibco, Waltham, USA) with 2% SDS and 50 mM DTT and heat denatured at 95 °C for 5 min. To avoid precipitation of SDS cool down was performed at room temperature. After equilibrating Amersham Protran Premium 0.45 μm nitrocellulose membranes (GE Healthcare, Solingen, Germany) with DPBS containing 0.1% SDS samples were filtered through the membranes by using a Minifold® II Slot Blot System (Whatman, Maidstone, UK). Slots were washed with one volume of DPBS before washing the whole membrane with TBS and blocking with 5% skimmed milk powder (Sigma-Aldrich, Munich, Germany) in TBS for 1 hour. Membranes were incubated overnight at 4 °C with primary antibodies (mouse-anti-Ataxin-3, 1:2500, clone 1H9, MAB5360, MerckMillipore, Darmstadt, Germany; rabbit-anti-Ubiquitin, 1:500, Z0458, Dako, Jena, Germany). After washing, the membranes were incubated with secondary antibodies (peroxidase affinePure donkey anti-mouse IgG H+L, 1:12000, 715-035-150, Jackson ImmunoResearch, Ely, UK; IRDye 800CW goat anti-mouse IgG (H+L) 1:1000, 926-32210, LI-COR, Bad Homburg, Germany; IRDye 800CW goat anti-rabbit IgG (H+L) 1:1000, 926-32211, LI-COR, Bad Homburg, Germany; goat anti-rabbit IgG H&L (HRP), 1:10.000, ab97051, Abcam, Cambridge, UK) for 1.5 hours at room temperature and detected as described in the section “Western blot analysis”.

### Time-Resolved Fluorescence Resonance Energy Transfer (TR-FRET) Immunoassay

TR-FRET immunoassay was used to measure total and expanded soluble Atxn3 protein levels in whole brain homogenates of 3- and 18-month-old mice. These immunoassays are based on an energy transfer of an excited donor fluorophore towards an acceptor fluorophore, which is only possible when both bind in close spatial proximity [27]. Terbium cryptate (tb) was used as donor fluorophore and d2 as acceptor fluorophore. To detect total soluble Atxn3 protein, 10 ng/μl anti-Atxn3-d2 (mouse-anti-Ataxin-3, clone 1H9, MAB5360, MerckMillipore, Darmstadt, Germany) and 0.5 ng/μl anti-Atxn3-(N-terminal)-tb (rabbit-anti-Ataxin-3, ab96316, Abcam, Cambridge, UK) were mixed in 1× detection buffer (in 50 mm NaH2 PO4, 400 mm NaF, 0.1% BSA, 0.05% Tween-20). To measure the amount of polyQ-expanded Atxn3, 3 ng/μl anti-polyQ-d2 (mouse-anti-Polyglutamine-Expansion Diseases Marker Antibody, clone 5TF1-1C2, MAB1574, MerckMillipore, Darmstadt, Germany) and 1 ng/μl anti-Atxn3-tb (mouse-anti-Ataxin-3, clone 1H9, MAB5360, MerckMillipore) were diluted in detection buffer. For both measurements, 5 μl of whole brain homogenates of 3- and 18-month-old mice were incubated with 1 μl of the respective antibody mix and incubated at 4 °C for 22 hours. Donor fluorescence was measured at 615 nm and acceptor fluorescence at 665 nm with EnVision multimode plate reader unit (PerkinElmer, Waltham, USA). For quantification, the acceptor to donor fluorescence ratio was calculated and corrected to total protein amount and negative control. The resulting fluorescence signal is proportional to the protein concentration.

### Immunohistochemistry, Immunofluorescence, and Microscopy

To obtain sections, mice were transcardially perfused with cold PBS and 4% paraformaldehyde (PFA) and the brains were post-fixed in 4% PFA overnight at 4 °C and subsequently embedded in paraffin.

For immunohistochemical and immunofluorescent staining paraffin-embedded brains were cut in 7 μm thick sagittal sections with the Leica RM2155 microtome (Leica, Wetzlar, Germany) and were rehydrated in xylene and a graded alcohol series using the Leica autostainer XL (Leica, Wetzlar, Germany). Microwave treatment with 10 mM sodium citrate and 10 mM citric acid for 15 min and washing with phosphate buffered saline (PBS) was performed.

For the immunohistochemical staining, the endogenous peroxidase was blocked by using 1.6% peroxidase (Sigma-Aldrich, Munich, Germany). After washing with PBS, sections were blocked in 5% normal goat serum (NGS) (Vector Laboratories, Burlingame, USA) in PBS supplemented with 0.3% Triton X-100 (Carl Roth, Karlsruhe, Germany) at room temperature for 45 min. After washing with PBS, sections were incubated with primary antibody mouse anti-Ataxin-3 (clone 1H9, 1:400, MerckMillipore, Darmstadt, Germany) diluted in PBS containing 15% NGS overnight at 4 °C in a humid chamber. After washing with PBS, biotinylated secondary antibody goat anti-mouse (1:200, Vector Laboratories, Burlingame, USA) were incubated on the sections for 1 hour at room temperature. In parallel, the avidin-biotin-complex (ABC) (Vector Laboratories, Burlingame, USA) was prepared and after secondary antibody incubation, ABC was incubated on the sections for two hours at room temperature. After washing with PBS, the substrate 3,3′-Diaminobenzidin (DAB, Sigma-Aldrich, Munich, Germany) was added to the sections and the reaction was stopped in distilled water when the desired degree of staining was reached. After dehydrating, the sections were mounted with CV Ultra mounting media (Leica, Wetzlar, Germany).

For the immunofluorescent staining deparaffinized sections were blocked in 5% NGS (Vector Laboratories, Burlingame, USA) in PBS supplemented with 0.3% Triton X-100 (Carl Roth, Karlsruhe, Germany) at room temperature for 45 min. After washing with PBS, sections were incubated with primary antibodies (mouse anti-Ataxin-3, 1:250, clone 1H9, MAB5360, MerckMillipore, Darmstadt, Germany; rabbit anti-IbaI, 1:1000, 019-19741, FUJIFILM Wako Chemicals Europe, Neuss, Germany; rabbit anti-Gfap, 1:500, Z0334, Dako Jena, Germany; rabbit anti-Olig2,1:500, AB9610, MerckMillipore, Darmstadt, Germany) diluted in PBS containing 15% NGS overnight at 4°C in a humid chamber. After washing in PBS, sections were incubated with secondary antibody goat anti-mouse-Alexa-Fluor488 (1:500, ab150113, abcam, Cambridge, UK) and goat anti-rabbit-Alexa-Fluor555 (1:500, A32732, ThermoFisher Scientifc, Waltham, USA) for 1 hour each, washed with PBS and mounted with VECTASHIELD antifade mounting medium with DAPI (H-1200, Vector Laboratories, Burlingame, USA). Sections were imaged within 7 days.

For cresyl violet staining, sections were treated with cresyl violet stain solution (0.1%, ab246816, abcam, Cambridge, UK) according to the manufacturer protocol and mounted with CV Ultra mounting media (Leica, Wetzlar, Germany).

Aggregate formation and state of the PCs were evaluated manually in two to three mice per genotype and time point and for both sexes. PCs were sorted into two classes, “intact” and “altered” PCs, by assessing size, shape and position within the PC layer. Further cells of the deep cerebellar nuclei (DCN) and the pontine nuclei were counted manually (only DCN) and with the automatic cell counting function of ImageJ (Wayne Rasband, NIH).

Imaging of the sections occurred with the Axioplan2 imaging microscope using an Axio-Cam HR color digital cam, a 20x Plan Neofluar and 63x Plan/Apochromat objective and the AxioVison SE64 Rel. 4.9 software (all Zeiss, Oberkochen, Germany).

Overview images were taken with the AxioScan.Z1 in 40-fold magnification and analyzed with the Zen2011 software (all Zeiss, Oberkochen, Germany).

### RNA Sequencing of Human and Mouse Cerebellar RNA

For mice, total RNA, microRNA, and DNA were extracted simultaneously using the AllPrep DNA/RNA/microRNA Universal Kit (Qiagen, Hilden, Germany) by using the manufacturer’s protocol. RNA was isolated from cerebellar tissue (*n* = 5 WT/WT and 304Q/304Q mice) at a pre-symptomatic (2 months of age) and symptomatic (12 months of age) time point. Samples with very high RNA integrity numbers (RIN > 8) were selected for library construction. A total of 100 ng of total RNA was subjected to polyA enrichment and cDNA libraries were constructed using the resulting mRNA and the TruSeq Stranded mRNA (Cat No 20020595, Illumina, San Diego, USA). Libraries were sequenced as paired-end 101 bp reads on a NovaSeq6000 (Illumina, San Diego, USA) with a depth of 16–38 million reads each. Library preparation and sequencing procedures were performed by the same individual and a design aimed to minimize technical batch effects was chosen.

For human samples, frozen tissue was homogenized in Qiazol Lysis Reagent (Qiagen Benelux, Venlo, The Netherlands). The total RNA including the microRNA fraction was isolated using a mi-RNeasy Mini kit (Qiagen Benelux, Venlo, the Netherlands) according to manufacturer’s instructions. RNA was isolated from six male SCA3 patients (mean age: 66.8 ± 7.7 years) and six male controls not affected by SCA3 (mean age: 64.3 ± 16.3 years). The human samples presented with a lower quality RNA integrity number (RIN > 4 and RIN < 7, DV200 >60 %) as well as a low quantity of RNA. Library preparation was performed by capture of sequence-specific coding RNA using 40 ng of total RNA and the TruSeq RNA Access Library Prep Kit (Cat No RS-301-2001, Illumina, San Diego, USA). Libraries were sequenced as paired-end 68 bp reads on a HiSeq2000 (Illumina, San Diego, USA) with a depth of approximately 25 - 100 million reads each.

### Analysis of RNA Sequencing

Quality of the RNA sequencing (RNA-seq) data was assessed using FastQC (v0.11.4) (http://www.bioinformatics.babraham.ac.uk/projects/fastqc) to identify sequencing cycles with low average quality, adaptor contamination, or repetitive sequences from PCR amplification before aligning reads with STAR (v2.5.4b) [28] against the Ensembl M. musculus GRCm38 and H. sapiens GRCh38 genome v91 allowing gapped alignments to account for splicing. Alignment quality was analyzed using samtools (v1.1) [29]. Normalized read counts for all genes were obtained using Rsubread (v2.0.0) and DESeq2 (v1.18.1) [30]. Transcripts covered with less than 50 reads were excluded from subsequent analyses leaving 12.108 (mouse) and 14.893 (human) genes for determining differential expression. The factorial design of the experiment was captured in a general linearized model defining gene expression as a function of genotype, age, and interaction of both. Significance thresholds were set to | log_2_ FC | ≥ 0.5 and FDR-adjusted *p*-value ≤ 0.05 as per Benjamini-Hochberg. Surrogate variable analysis (sva, v3.26.0) [31] was used to minimize unwanted variation between samples. Gene-level abundances were derived from DESeq2 as normalized read counts and used for calculating the log2-transformed expression changes of the expression heatmap and centroids. Ratios were relative to mean expression in WT/WT2m. Read counts obtained from Rsubread also went into calculating nRPKMs (normalized Reads Per Kilobase per Million total reads) as a measure of relative gene expression as motivated before [32]. Orthologous genes between mouse and human were determined with the biomaRt package on the v91 of the Ensembl genome annotations. Cell type-specific expression data for mouse and human were adapted from Zhang, Chen [33] and Kuhn, Kumar [34], respectively. WebGestalt was employed to identify overrepresented molecular functions among Gene Ontology terms [35]. KI raw sequencing files are available through GEO under accession number: GSE145613. Human RNA-seq data set has been deposited at the European Genome-phenome Archive (EGA), which is hosted by the EBI and the CRG, under the accession number: EGAS00001004241.

### Quantitative Reverse Transcription PCR (qRT-PCR)

qRT-PCR was performed for validation of the differentially expressed genes *Atxn3, Igfbp5, Il20rb, Il33*, and *Syndig1l* as well as for the oligodendrocyte markers *Mobp, Plp1*, and *Mbp*. 500 ng purified RNA was transcribed into cDNA using QuantiTect Reverse Transcription Kit (Qiagen, Hilden, Germany). For each gene of interest, 2 μl of diluted cDNA (1:20) were added to SYBR Green PCR Master Mix (Qiagen, Hilden, Germany) and 10 μM of both forward and reverse primer (Primer Sequences Table 1). qRT-PCR was run on the LightCycler 480 II (Roche, Mannheim, Germany). The relative gene expression was calculated by normalization to housekeeping genes *Sdha, Pdh, mActb* and *Tbp* (Primer Sequences Table 1).

### Statistical Analysis

For statistical analysis, GraphPad Prism 6.0 for Windows (GraphPad Software Inc., San Diego, USA) was used. Significance of datasets was determined using two-tailed Student’s *t*-test comparing either WT/WT animals with each KI group (significances marked by *) or the mice of the KI lines WT/304Q and 304Q/304Q (marked by #), respectively.

For behavior analysis at single time points Welsh Correction was applied. For longitudinal studies Tukey’s multiple comparisons test was applied.

Repeated RotaRod tests over multiple time points were tested for normality using Shapiro-Wilk test. Afterwards, RotaRod performance data were analyzed using two-way ANOVA adjusted for sex and body weight as covariable using IBM SPSS Statistics version 27.

*P* values with less than 0.05 were considered statistically significant with */# *p* < 0.05, **/## *p* < 0.01, and ***/### *p* < 0.001. All values are shown as mean ± standard derivation, SEM.

Enrichments for cell types are based on Fisher’s exact tests (R stats package v3.6.2). Shifts in cell-type composition were assessed using Mann Whitney *U*-tests (R stats package v3.6.2).

## Results

### Zinc-Finger Generated SCA3 KI Line Express Expanded Atxn3 with an Interrupted CAACAGCAG Repeat

**Fig. 1:**
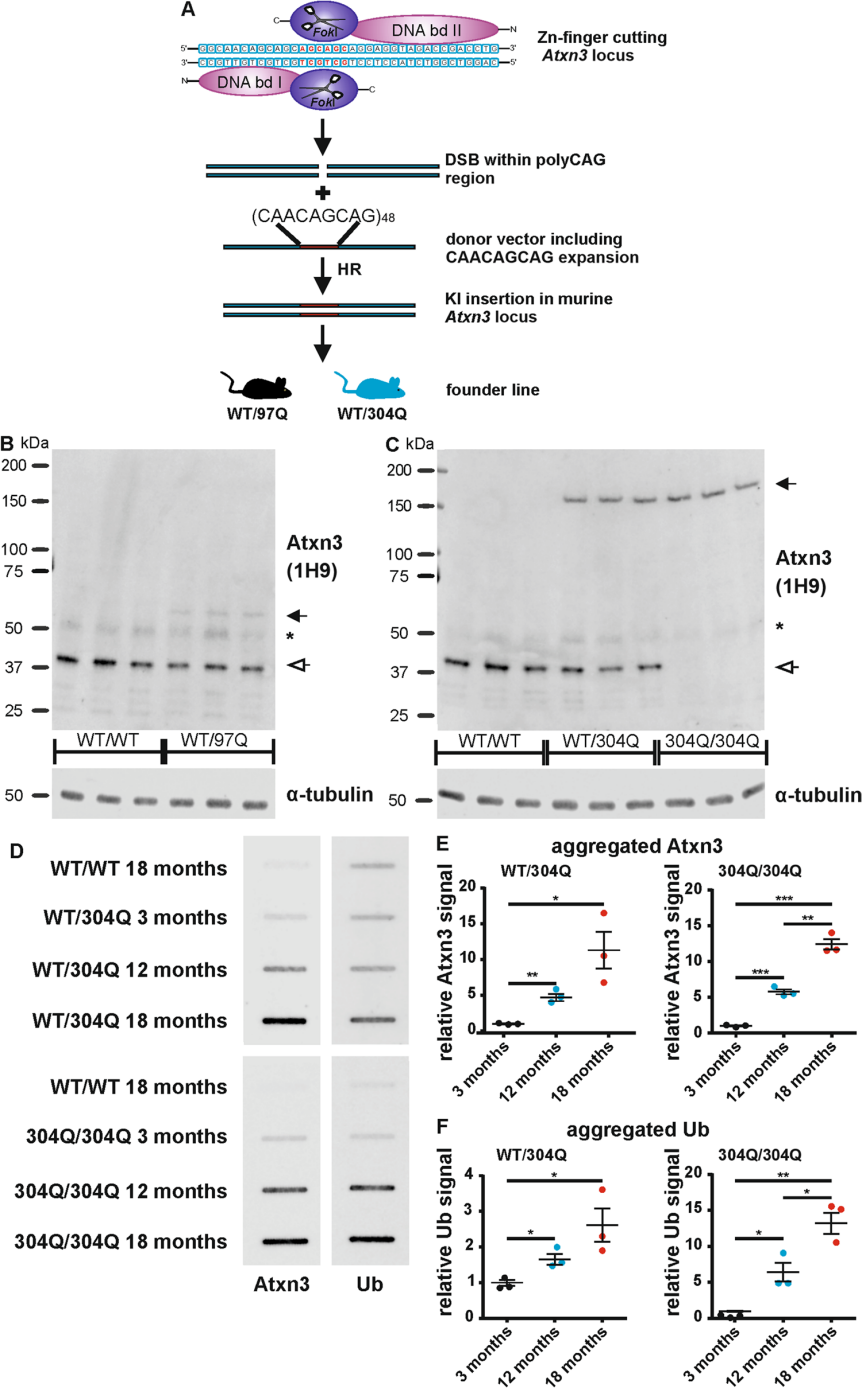
Zn-finger generated SCA3 KI mice showed aggregated Atxn3 and Ub**.** (**A**) Schematic representation of KI generation. Zn-finger nuclease DNA binding domains (bd) recognize sequences in the mouse genome to introduce a double-strand break (DSB) in the mouse *Atxn3* CAG repeat region. Using a donor vector with (CAACAGCAG)_48_ as template for homologous recombination (HR) two mouse lines containing 97 or 304 CAA/CAG repeats in the *Atxn3* locus were generated. (**B**+**C**) Western Blot analyses of whole brain lysates showed the expression of the polyQ-expanded and non-expanded Atxn3 in the heterozygous KI line WT/97Q mice (**B**) and in the heterozygous and homozygous KI lines WT/304Q and 304Q/304Q (**C**) with 3 months of age . (**D**-**F**) In whole brain homogenates of WT/304Q and 304Q/304Q mice, a significant increase in the amount of Atxn3 positive insoluble aggregates was observed over time by filter retardation assay (**D** + **E**). In WT/304Q and 304Q/304Q aggregated material was also stained positive for Ub, and Ub signal was increasing over time (E-F). *n* = 3, both sexes, (**B**-**C**) filled arrow = polyQ-expanded Atxn3, unfilled arrow = non-expanded Atxn3; * IgG artefact; (**E**-**F**) filter retardation assays signal values normalized to signal of 3-month-old WT/304Q or 304Q/304Q mice, respectively. Two-tailed Student’s *t*-test, * *p* < 0.05, ** *p* < 0.01, *** *p* < 0.001. α-tubulin shown as loading control

Our goal was the generation of a new SCA3 KI mouse line with a hyper-expanded polyQ stretch in the murine *Atxn3* gene under control of all endogenous regulatory elements to trigger a neurological phenotype comparable to human SCA3 patients. Thus, we used Zinc-finger (Zn-finger) technology [25] to introduce an interrupted CAACAGCAG expansion into the murine *Atxn3* locus in mice with C57BL/6 background (Fig. 1A). Here, the DNA binding domains I and II recognized sequences flanking the endogenous murine sequence CAA(CAG)_5_ and introduced a double-strand break (DSB). For homologous recombination (HR), we used a donor vector including the interrupted sequence (CAACAGCAG)_48_ flanked with 800 bp of the up- and downstream region of *Atxn3*. Due to the repetitive sequence in the donor vector, we obtained two founder lines one containing 97 CAACAG repeats and one containing 304 CAACAG interrupted repeats, leading to the corresponding length of polyQ-expansion in the Atxn3 protein (in the following called WT/97Q and WT/304Q mice) (Fig. 1A). WT/97Q mice serve merely as a control group, since they demonstrated comparable features as shown for the SCA3 KI mouse lines mice from Ramani [20] and Switonski [22]. To analyze a potential gene dosage effect, homozygous mice (304Q/304Q) were bred as well. All genotypes were viable, fertile, and showed no signs of reduced survival. Intergenerational stability was tested regularly by fragment length analysis and no signs of an increase or decrease in the CAACAG tract was so far detected since 2015. Protein analysis of the WT/97Q (Fig. 1B), WT/304Q and 304Q/304Q (Fig. 1C) mice confirmed the expression of non-expanded (WT) and expanded Atxn3 in whole brain lysates of the WT/97Q, WT/304Q and 304Q/304Q line at an early (3 months of age) and late (18 months of age) time point (data not shown). Non-expanded Atxn3 was detected with a molecular weight of 42 kDa in WT/WT, WT/97Q and WT/304Q mice (Fig. 1B + C, unfilled arrow). The expanded Atxn3 was detected with a molecular weight of about 60 kDa in the WT/97Q (Fig. 1B, filled arrow) mice and with 180 kDa in WT/304Q and 304Q/304Q mice due to the increased polyQ size in the Atxn3 protein. Analysis of whole brain lysates of homozygous 304Q/304Q mice showed expression of expanded Atxn3 and complete loss of non-expanded Atxn3 (Fig. 1C). No differences between male and female mice were observed.

Further, protein analysis confirmed the expression of expanded Atxn3 in a wide range of tissues such as heart, lung, liver (Online Resource SI 1A), kidney, spleen and muscle (Online Resource SI 1B). Due to the low expression in certain tissues and other tissue differences, two different anti-Atxn3 antibodies had to be used to detect expanded Atxn3 in all tissues (Online Resource S1 1A + 1B)

### Accumulation of Ub-Positive Aggregates Atxn3 in SCA3 KI Mice with 304Q

One hallmark of SCA3 is the formation of Ub-positive Atxn3 protein aggregates [36]. To demonstrate this feature in our KI mice, we investigated the formation of detergent-insoluble aggregates in whole brain homogenates by filter retardation assay. For KI mice expressing expanded Atxn3, a massive increase of Atxn3-positive aggregates was detectable within 18 months (Fig. 1D + 1E). Specifically, we observed an increase of 4.6-fold in the first 12 months and a final increase of 11.3-fold at 18 months of age for the number of Atxn3-positive aggregates in WT/304Q mice compared to 3 months (Fig. 1D + E). In 304Q/304Q, an increase of 5.8-fold of Atxn3-positive aggregates was observed within 12 months, and an increase of 12.4-fold at 18 months of age (Fig. 1D + E). In both cases, the formed aggregates were Ub-positive (Fig. 1D), showing mild ubiquitination already at 3 months of age that kept increasing over time. In fact, for the heterozygous WT/304Q mice, we observed an increase of 1.7-fold in the first 12 months and an increase of 2.6-fold at 18 months of age (Fig. 1F). This effect was drastically stronger in homozygous 304Q/304Q mice, where the increase of Ub-positive aggregates was 6.4-fold within 12 months and 13.2-fold at 18 months of age (Fig. 1F), respectively. We did not observe differences in the formation of aggregates between male and female animals.

To demonstrate, that a hyper-expansion of the polyQ tract is necessary to trigger aggregate formation within the murine gene by the protein expression machinery, we investigated the presents of aggregates in heterozygous or hemizygous SCA3 models, including our new KI mouse models expressing either 97Q or 304Q and the YAC84Q mice [19] by filter retardation assay. In the WT/97Q mice we could detect neither Atxn3- nor Ub-positive aggregates with either 3 or 18 months of age (Online Resource SI 1C – S1E). The YAC84Q mouse model, which is expressing the expanded human Atxn3 gene next to the non-expanded murine Atxn3, presented Atxn3 –aggregates already in 3-month-old mice, but no further increase in 18-month-old mice s (Online Resource SI C + 1D). Highest amounts of Atxn3 aggregates were observed in the 18-month-old WT/304Q mice in this experiment (Online Resource SI 1C+ D). Ubiquitination of the aggregates was only observed in the WT/304Q mice with 18 months of age and only slightly in hemizygous YAC84Q mice at the age of 18 months (Online Resource SI 1C + S1E).

### Aggregate Formation Occured in SCA3 Associated Brain Regions Accompanied by Purkinje Cell Loss

**Fig. 2:**
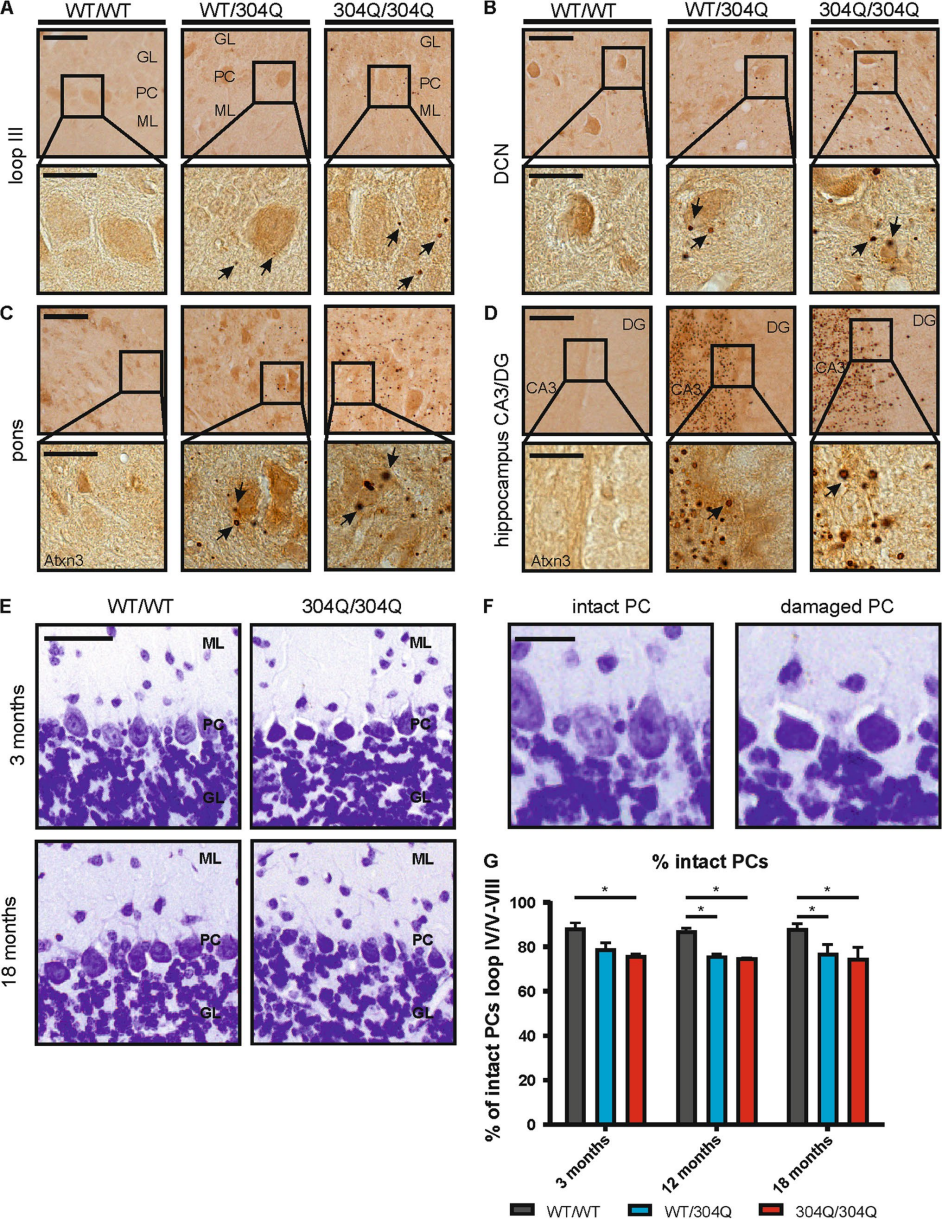
Aggregated mutant Atxn3 in 18-month-old SCA3 KI mouse brain. Immunohistochemical (IHC) staining using Atxn3-specific antibody (clone 1H9) showed aggregate formation in WT/304Q and 304Q/304Q mice. (**A**–**D**) At 18 months, increased diffuse nuclear staining and massive formation of aggregates were detectable in both WT/304Q and 304Q/304Q mice. Most aggregates were found in the DCN (**B**), the pons (**C**), and the hippocampal CA3 region (**D**). (**E**–**G**) The number of intact PCs in the cerebellar loops IV/V to VIII were determined with cresyl violet staining in sections of 3- and 18-month-old mice (**F**) and revealed a significant reduction of intact PCs in 304Q/304Q mice with 3, 12 and 18 months of age and in WT/304Q mice with 12 and 18 months of age compared to WT/WT. (**E**) Representative magnifications of intact and damaged PCs. (**A**–**E**) Scale bar = 50 μm, inset scale bar = 20 μm, (**F**) scale bar 20 μm. Atxn3 positive aggregates are indicated by arrows. (**A**–**D**) *n*=3, both sexes, (**E**–**G**) *n* = 2, both sexes, Tukey’s multiple comparisons test, *or # *p* < 0.05; GL = granular layer, PC = Purkinje cells, ML = molecular layer; DG = dentate gyrus; DCN = deep cerebellar nuclei

Having observed a massive formation of detergent insoluble aggregates in the filter retardation assays, we tested which brain areas were most severely affected in our 304Q KI model. Immunohistochemical staining (IHC) for Atxn3 in 3-month-old animals already showed the formation of aggregates in the hippocampus of WT/304Q mice, and in loop III of the cerebellum, the deep cerebellar nuclei (DCN), the pons, and the hippocampus in 304Q/304Q mice (Online Resource SI 2A - S2D). All these areas have been reported to be vulnerable brain regions in SCA3 patients [9, 10]. With 18 months of age, we observed aggregates in all brain areas in both WT/304Q and 304Q/304Q mice (Fig. 2A–D). While the number of aggregates stayed at a moderate level in the cerebellar loop III (Fig. 2A), high numbers of aggregates were detected in the DCN, pons, and hippocampus (Fig. 2B–D). Similar results were already obtained in an NfL biomarker study comparing NfL release during neurodegenerative processes in SCA3 patients and the WT/304Q KI mouse model [11]. In WT/97Q no Atxn3-positive aggregates were found in any investigated brain area (data not shown).

As Atxn3 is a ubiquitous expressed protein, we quantified several brain areas to detect the extend of the affected brain regions. A summary of this quantification is depicted in Table 2. At later time points, we did not find any brain region free of Atxn3 aggregates. These findings are in line with described aggregate formation found in post-mortem human SCA3 brain samples as described by Wilke et al. [11].

**Table 2:** Development and distribution of Atxn3 aggregates in the brains of WT/WT, WT/304Q and 304Q/304Q mice within 18 months *n* =3, both sexes

	WT/WT			WT/304Q			304Q/304Q		
	3 months	12 months	18 months	3 months	12 months	18 months	3 months	12 months	18 months
**Brainstem**	-	-	-	+	++	+++	+	+++	+++
**Cerebellum**									
DCN	-	-	-		+	+	(+)	++	+++
GL	-	-	-	-	+	++	(+)	++	++
ML	-	-	-	(+)	(+)	(+)	-	+	+
PC	-	-	-	-	(+)	(+)	-	+	+
**Cortex**	-	-	-	-	++	+++	++	+++	+++
**Hippocampus**									
CA1	-	-	-	(+)	+	++	+	++	+++
CA3	-	-	-	-	++	++	++	+++	+++
DG	-	-	-	(+)	+++	+++	+++	++++	++++
**Hypothalamus**	-	-	-	+	+	++	+	++	+++
**Midbrain**	-	-	-	(+)	++	+++	+	+++	+++
**Olfactory bulb**	-	-	-	(+)	++	+++	++	+++	+++
**Pons**	-	-	-	+	++	++	+	+++	+++
**Thalamus**	-	-	-	(+)	+	++	+	++	+++

*CA* cornu ammonis; *DCN* deep cerebellar nuclei; *DG* dentate gyrus; *GL* granular layer; *ML* molecular layer; *PC* Purkinje cells. + few number of aggregates, ++ intermediate number of aggregates, +++ high number of aggregates, ++++ excessive number of aggregates

The massive increase in aggregates in certain brain areas (e.g., hippocampus) was even visible in sagittal overview images of brains in 18-month-old 304Q/304Q KI mice (Online Resource SI 3).

Further, we investigated the state of the cerebellar PCs, a cell type affected in SCA3 patients [37]. Staining with cresyl violet, a stain for neuronal cell bodies, showed a significant reduced number of intact PCs in cerebellar loop IV/V to loop VIII in 3-, 12-, and 18-month-old 304Q/304Q mice (Fig. 2E + G). Fig. 2F shows representative images of intact and damaged PCs. Damaged PCs were stained a darker shade of violet, as the dye could penetrate the cells more easily. Further the cells are less round than intact ones, which makes them easy recognizable. For the WT/304Q we observed a tendency towards a reduction already with 3 months of age and detected a significant reduction in 12- and 18- months old mice (Fig. 2G). Investigating the numbers of affected PCs in the hemizygous YAC84Q model, we also saw a trend towards a reduction in 3-month-old mice and a significant reduction with 18 months of age, indicating a similar effect as in the WT/304Q KI mice (Online Resource SI 2E + S2F). We also counted the DCNs and pontine nuclei as described in Wilke et. al [11] without detecting any significant changes in the cell numbers.

### Impaired Physical Condition and Body Weight Reduction in Male Mice

**Fig. 3:**
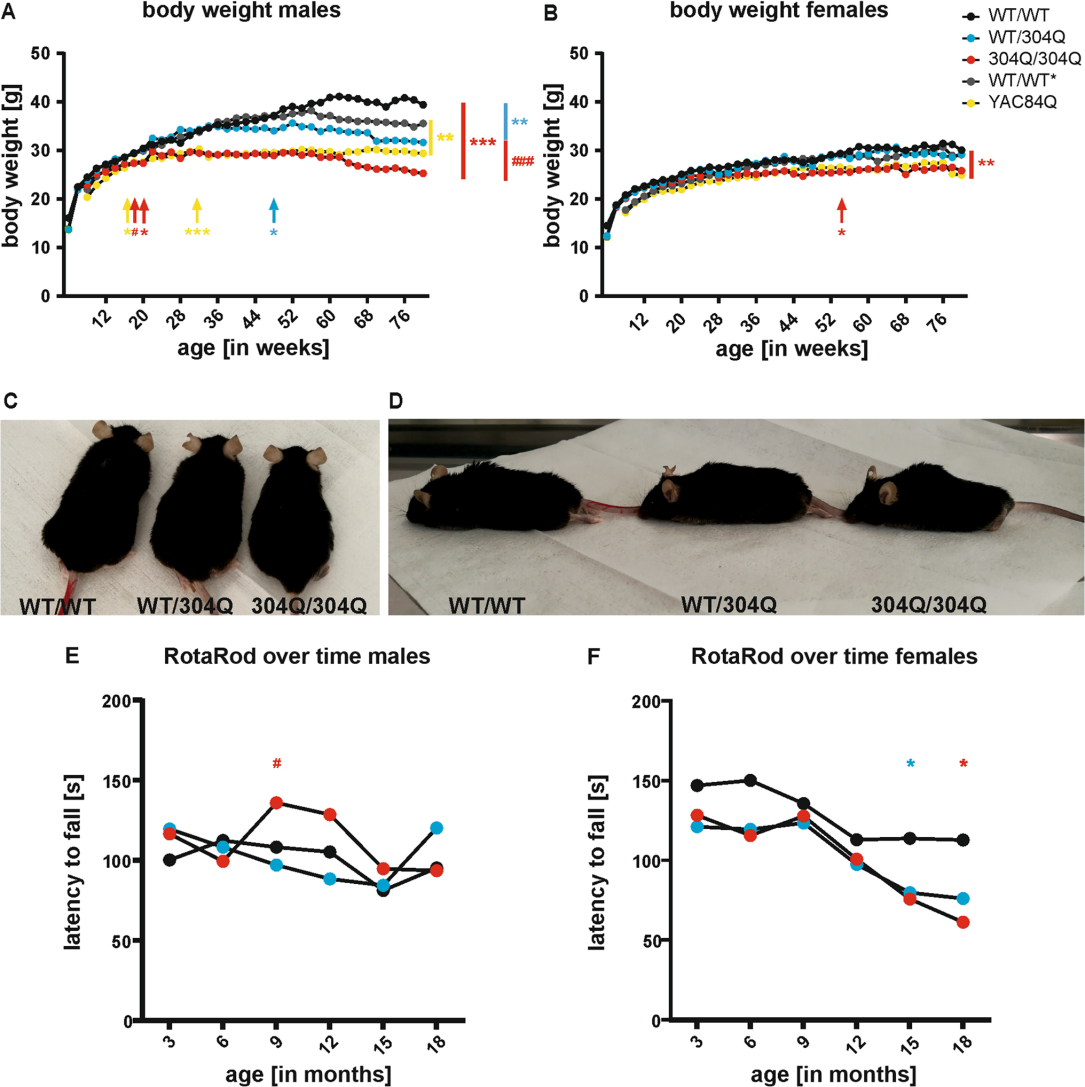
KI mice showed size, posturing, and body weight alterations accompanied by balance and gait instability. (**A**) Body weight of male WT/304Q and 304Q/304Q mice is significantly reduced compared to controls after 18 months. YAC84Q mice and their controls (WT/WT*) are shown for comparison. Body weight of YAC84Q mice is comparable to 304Q/304Q in early and intermediate ages and in between of WT/304Q and 304Q/304Q towards later stages. (**B**) In female mice only 304Q/304Q mice showed a reduction in body weight. Female YAC84Q mice presented with comparable body weight to 304Q/304Q mice. Beginning of significant differences are indicated by arrows. (**C**+**D**) Alterations in size (**C**) and posturing (**D**) were well recognizable in 18-month-old male KI mice. (**E**) 304Q/304Q male mice showed better coordination compared to WT/WT littermates at 9 and 12 months of age. WT/304Q males performed better at 18 months of age than their 304Q/304Q littermates. (**F**) WT/304Q and 304Q/304Q female mice tend to perform worse than their WT/WT littermates at all measured time points, reaching significance with 15 and 18 months of age. *n* = 6–8 mice per genotype and sex, (**A**–**B**) WT/WT, WT/304Q, 304Q/304Q males *n* = 7–8, females *n* = 6–7, WT/WT*, YAC84Q males *n* = 6–24, females *n* = 4–23; two-tailed Student’s *t*-test with Welsh correction for each time point, (**E**–**F**) Shapiro-Wilk test and two-way ANOVA adjusted for sex and body weight as covariable using IBM SPSS Statistics version 27, males *n* = 7–8, female *n* = 6–7; *or # *p* < 0.05, ** *p* < 0.01, *** or ### *p* < 0.001, * comparison WT/WT to KI lines, # comparison WT/304Q to 304Q/304Q, black = WT/WT, cyan = WT/304Q, red = 304Q/304Q , grey = WT/WT* (respective control to YAC84Q), yellow = YAC84Q

As the body mass index (BMI) is known to be reduced in SCA3 patients compared to controls [38] and to reversely correlate with the length of the expanded polyQ [39, 40], we investigated whether our SCA3 KI mice display differences in body weight compared to their WT/WT littermates. Therefore, body weight of the mice was measured every other week. Separating cohorts by sex revealed that male WT/304Q and 304Q/304Q mice (Fig. 3A) were affected earlier and more severely by body weight reduction than female mice (Fig. 3B). 304Q/304Q males showed significant weight reduction compared to their heterozygous WT/304Q littermates already at 18 weeks of age and to their WT/WT littermates at 20 weeks of age. Heterozygous WT/304Q male mice had a significant reduction in body weight compared to WT/WT animals starting at 48 weeks of age. At the last measurement point in our study (at 18 months of age), WT/304Q mice and 304Q/304Q mice had an approximate weight reduction of 24% and 36%, respectively, when compared to WT/WT male mice. Comparing the body weights of male WT/304Q and 304Q/304Q mice to that of hemizygous YAC84Q mice, we observed that up to 62 weeks the YAC84Q and 304Q/304Q mice showed similar body weights and a significant reduction compared to their WT/WT controls. In the later stages, we observed a further reduction of the body weight only in the 304Q/304Q mice, while the hemizygous YAC84Q mice stagnated on the same level, between WT/304Q and 304Q/304Q mice (Fig. 3A). In females, we observed a significant reduction in body weight only in 304Q/304Q mice compared to WT/WT littermates starting at 40 weeks of age and compared to their WT/304Q littermates at 42 weeks of age (Fig. 3B), but these differences did not reach significance after 58 weeks . At the last time point of investigation, the 304Q/304Q mice had an approximate 14% reduction in their body weight compared to WT/WT littermates. The female hemizygous YAC84Q mice and the 304Q/304Q mice presented with similar body weights, however the differences between hemizygous YAC84Q and their WT/WT* littermates did not reach significance (Fig. 3B). Neither male nor female WT/97Q mice were significantly different in their body wight development when compared to their WT/WT littermates (data not shown).

Moreover, a reduction in body weight did not represent the only physical change in our newly generated 304Q KI mouse line. Differences in body size (Fig. 3C) and posturing (Fig. 3D) became apparent for WT/304Q and 304Q/304Q mice. In both, we observed a reduced body size and enhanced hunchback development. Both findings were more pronounced in males (Fig. 3C + D) and less or not at all detectable in female mice (data not shown). In WT/97Q mice no such physical changes were observed.

### Female Mice Present with Impaired Coordination and Stability Phenotype While Gait Analysis Was Changed in Both Sexes

Changes in body weight also influence the coordination and balancing phenotype of the KI mice. RotaRod analysis conducted to address these features revealed different results for male and female mice. In male mice, no differences between WT/WT and WT/304Q mice were observed, while the 304Q/304Q mice performed better at intermediate time points (9 and 12 months of age) and became comparable to WT/WT and WT/304Q mice in later time points (15 and 18 months) (Fig. 3E). In female mice (Fig. 3F), WT/304Q and 304Q/304Q showed a tendency for coordination deficits already in young mice (3-month-old) and became significant at the last investigated time point (18-month-old). A detailed presentation of RotaRod analysis for males and females at baseline (3 months) and last measured time point (18 months) demonstrated no coordination differences in young mice (3 months) in neither males (Online Resource SI 4A) nor females (Online Resource SI 4B). In old male mice (Online Resource SI 4C), we observed a tendency for coordination differences in 304Q/304Q mice compared to the WT/304Q mice, while in old female mice (Online Resource SI 4D) we found significantly impaired coordination in WT/304Q and 304Q/304Q mice compared to their WT/WT littermates. We also performed RotaRod performance with the hemizygous YAC84Q mice and their WT/WT* littermates (Online Resource SI 4E + S4F). For the hemizygous male YAC84Q mice (Online Resource SI 4E) we observed no differences between genotypes. For visualization we plotted the results of the WT/304Q mice in the same graph, to indicate their worse performance in this test, although performed independently. Hemizygous female YAC84Q mice on the other hand, tended to fall off the rod earlier than their WT/WT* littermates and the female WT/304Q at the time points 3 to 12 months, but no significances were observed. RotaRod analyses for the 97Q KI mice line was not performed. Differences in body weight were considered as covariable in all statistical analysis of the RotaRod test.

**Fig. 4:**
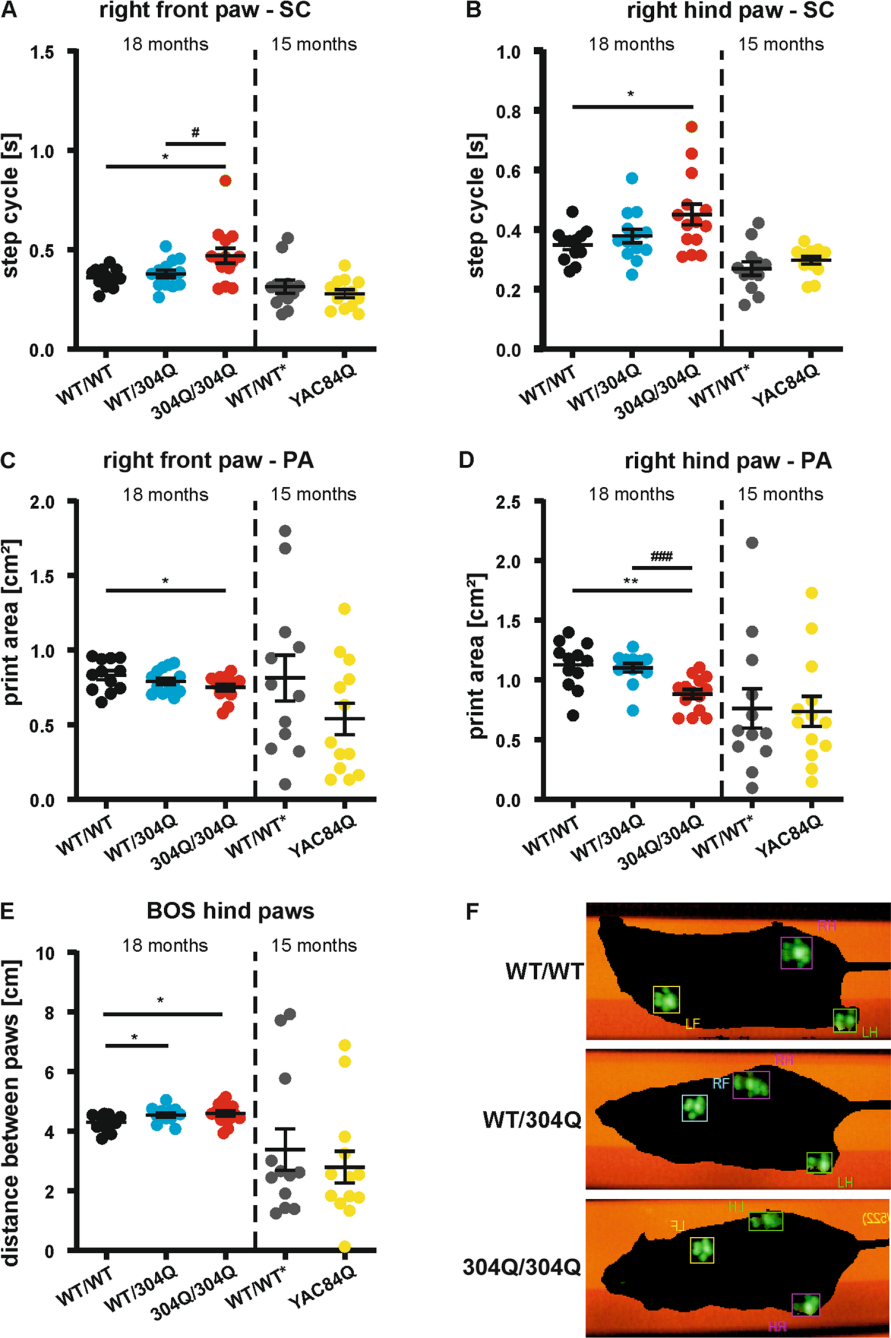
Altered paw positioning (right side) in 18-month-old WT/304Q and 304Q/304Q KI mice. (**A**-**B**) Step cycle (SC) was increased for 304Q/304Q mice in the right front paw compared to WT/WT and WT/304Q mice (**A**) and in the hind paw (**B**) in comparison to WT/WT littermates. (**C**–**D**) Print area (PA) of the right front (**C**), and hind paw (**D**) was significantly reduced in 304Q/304Q mice. For the hind paw, this reduction was also significantly different to WT/304Q mice. (**E**) Base of support (BOS) was significantly increased in KI mice compared to WT/WT mice. (**F**) Visualization of the footprint analysis showed an increase in BOS for WT/304Q and 304Q/304Q mice. No differences in any of the described parameters were observed between YAC84Q mice and their WT/WT* controls with 15 months of age. *n* = 12–16 mice per genotype including both sexes, two-tailed Student’s *t*-test with Welsh correction, *or # *p* < 0.05, ** *p* < 0.01, *** or ### *p* < 0.001, * comparison WT/WT to KI lines, # comparison WT/304Q to 304Q/304Q, black = WT/WT, cyan = WT/304Q, red = 304Q/304Q, grey = WT/WT* (control to YAC84Q), yellow = YAC84Q

Gait analysis, which was not as strongly affected by the body weight, showed significant differences in several gait parameters determined by the Catwalk (Noldus) gait analysis system in 18-month-old mice for the pooled cohort without sex separation. For step cycle (SC) analysis, the time it takes a mouse to remove the paw from the surface and to place it back was measured, and we observed for the right front paw (Fig. 4A) an increased SC in 304Q/304Q mice compared to their WT/WT and WT/304Q littermates. For the right hind paw (Fig. 4B) both lines, WT/304Q and 304Q/304Q, showed a significantly increased SC time compared to WT/WT mice. The print area (PA) describes the area of the paw that touches the surface during walking. For the right front paw (Fig. 4C) we observed reduced PA in 304Q/304Q mice compared to WT/WT mice and for the right hind paw (Fig. 4D), the PA of 304Q/304Q mice were significantly reduced to WT/WT and WT/304Q mice, respectively. Additionally, the base of support (BOS) of the hind paws (Fig. 4E), describing the distance between the left and right hind paw and giving an indication of the balancing capabilities of the mice, was investigated. Here, we observed a significant increase in the BOS for WT/304Q and 304Q/304Q mice, indicating the need of a wider stand for stabilization compared to their WT/WT littermates. The BOS of the front paws did not reveal any differences between the three genotypes (Online Resource SI 5E). The results for the right and left paws were comparable, but not identical, therefore only the right side is presented. The results for the left paws can be found as supplemental material (Online Resource SI 5). Representative images of the Catwalk quantification (Fig. 4F) demonstrate a reduced PA for the 304Q/304Q mice and a decreased BOS for WT/304Q and 304Q/304Q.

Sex separation of SC, PA, and BOS demonstrated similar tendencies in males and females, but in most cases did not reach significance potentially due to reduced animal numbers. The results for the sex separated right side and the BOS of the hind paws are shown in Online Resource SI 6 and for the left side and the BOS of the front paws in Online Resource SI 7. Further parameters, which describe ataxic gait parameter, investigated by Catwalk gait analysis system, that did not show other significant results are presented in Online Resource SI 8. We would like to emphasize that in a later study it was possible to detect first abnormalities in walking behavior in the 304Q/304Q mice with 9 months of age, when analyzing their ability to walk on an elevated beam by the “DeepLabCut” software based on deep learning networks [41].

For all gait related parameters investigated in this study, we also present the results of the hemizygous YAC84Q mice compared to their WT/WT* littermates with 15 months of age (latest investigated time point). None of the significantly altered parameters in the WT/304Q or 304Q/304Q mice were affected in the YAC84Q mice. However, most of the parameters showed a similar tendency and it is possible that some of them would reach significance if analyzed at the age of 18 months. Gait analyses of the 97Q KI mice line did not reveal any differences.

### RNA-seq Revealed Differences in Cerebellar Gene Expression Starting at 2 Months of Age

**Fig. 5:**
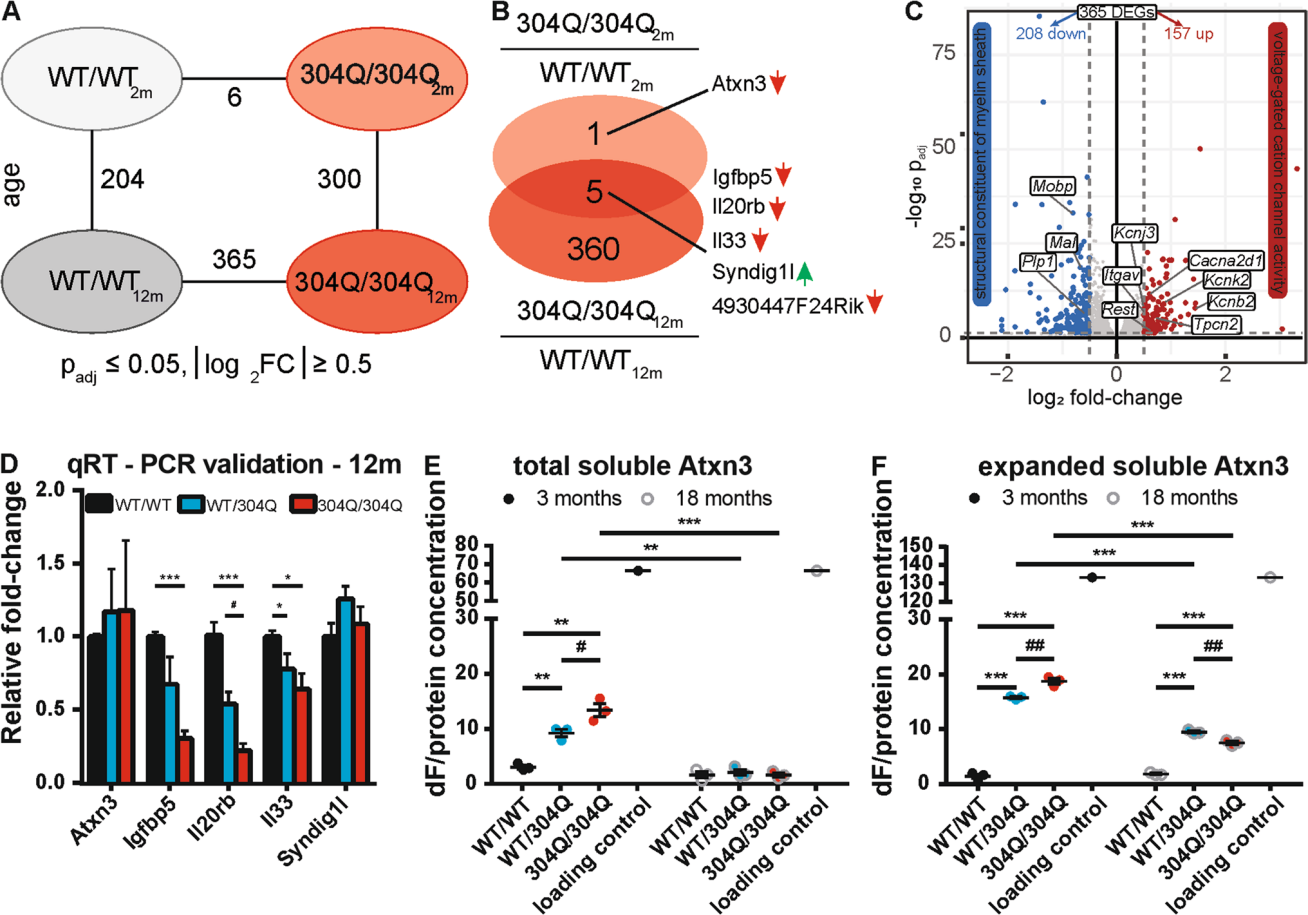
Altered gene and protein expression in SCA3 KI mice. (**A**) Total RNA from cerebellar tissue was isolated from 2- and 12-month-old WT/WT and 304Q/304Q mice for RNA-seq to investigate transcriptomic changes. Primary contrasts along the genotype and age dimension showed six DEGs in 2-month and 365 DEGs in 12-month-old animals. (**B**) Genotype effect revealed five differentially expressed genes common in 2- and 12-month-old 304Q/304Q KI mice. (**C**) Volcano plot of the 365 DEGS separated by magnitude and significance values of gene expression changes identified in cerebellar samples of 304Q/304Q mice compared to WT/WT littermates. Majority of the downregulated genes associate with GO term “structural constituent of myelin sheath”, while upregulated genes associate with “voltage-gated cation channel activity. (**D**) qRT-PCR validation in cerebellar RNA samples of 12-month-old mice confirmed significant downregulation of *Igfbp5, Il20rb* and *Il33* in 304Q/304Q KI mice. No significant differences for *Atxn3* were detected. (E-F) Protein expression in the cerebellum of total soluble Atxn3 (**E**) showed a significant increase in young WT/304Q and 304Q/304Q mice compared to WT/WT littermates. For 304Q/304Q mice, the increase is also significant compared to WT/304Q mice. With 18 months of age, no differences between the genotypes were observed. In 3-month-old mice the expanded soluble Atxn3 (**F**) was quantified only in WT/304Q and 304Q/304Q mice and was increased in 304Q/304Q mice compared to WT/304Q mice. With 18 months of age, the amounts of expanded soluble Atxn3 were reduced in WT/304Q and 304Q/304Q compared to the levels with 3 months of age. Both, total soluble and expanded soluble Atxn3 were reduced in disease context compared to the same genotypes with 3 months of age. RNA-seq *n* = 5, males only; qRT-PCR *n* = 3, males only; protein analysis n=3, both sexes; two-tailed Student’s *t*-test * or # *p* < 0.05, ** or ## *p* < 0.01, * comparison WT/WT to WT/304Q and 304Q/304Q, # comparison WT/304Q to 304Q/304Q, 2m/12m = 2/12 months old animals

RNA-seq of cerebellar samples of pre-symptomatic (2 months) and symptomatic (12 months) 304Q/304Q mice and their age-matched WT/WT littermates showed differential expression of six genes at the pre-symptomatic time point and 365 differentially expressed genes (DEGs) at the symptomatic time point. Along the age dimension, 204 genes were differentially expressed comparing 2- and 12-month-old WT/WT mice, and 300 genes comparing 2- and 12-month-old 304Q/304Q mice (Fig. 5A). Overlapping DEGs for pre-symptomatic and symptomatic 304Q/304Q mice, identified five out of six genes (*Igfbp5, Il20rb, Il33, Syndig1l, 4930447F24Rik*) already affected at the pre-symptomatic stage, being still differentially regulated in the symptomatic stage, indicating an early onset in differential expression that was still observed with 12 months of age over time (Fig. 5B). Importantly, the differential expression of *Il33* was already described earlier for YAC84Q mice [42, 43]. The total of 365 genes differentially expressed in the symptomatic 304Q/304Q mice compared to their WT/WT littermates were enriched for several Gene Ontology (GO) terms among which voltage-gated cation channel activity was most significant (Online Resource SI 9C). When analyzing up- and downregulated genes separately, this term was again most significant (p_adj_ 6E-05) for the 157 upregulated DEGs, whereas structural constituent of myelin sheath was most significantly enriched (p_adj_ 1E-05) among the 209 downregulated DEGs (Fig. 5C). To further confirm changes in gene expression seen by RNA-seq, we validated the expression changes of the overlapping genes *Igfbp5, Il20rb, Il33*, and *Syndig1l*, by qRT-PCRs for both time points in WT/WT, WT/304Q, and 304Q/304Q cerebellar samples. In 2-month-old mice changes in gene expression did not reach significances using qRT-PCR (Online Resource SI 9B). In the 12-month-old mice (Fig. 5D), we confirmed downregulation of *Igfbp5, Il20rb* and *Il33* in 304Q/304Q cerebellar samples compared to WT/WT littermates. *Il20rb* was also significantly reduced in 304Q/304Q samples compared to heterozygous littermates and *Il33* in WT/304Q compared WT/WT. For all three downregulated genes, the heterozygous WT/304Q samples showed an intermediate level between those of WT/WT and 304Q/304Q. Differences in *Syndig1l* gene expression could not be confirmed by qRT-PCR.

Next, we investigated, whether we could confirm the changes we have seen on RNA level also on protein level. However, we observed only marginal changes between genotypes of the animals in cerebellar samples (Online Resource SI 9D–S9G) at both investigated time points. Igfbp5 was not detectable in 3- and 12-months-old mice independent from the genotype as well as in prenatal rat tissue used as control (data not shown). For *Atxn3* itself we observed a downregulation in 2-month-old 304Q/304Q mice in our RNA-Seq data, while its gene expression stayed comparable in 12-month-old WT/WT and 304Q/304Q mice to levels of 2-month-old 304Q/304Q mice (Online Resource SI 9A). This was also observed by qRT-PCR in 2-month-old mice (Online Resource SI 9B), where we observed a tendency towards a reduction in the *Atxn3* level in the WT/304Q and 304Q/304Q mice compared to their WT/WT littermates. To specifically analyze full-length wildtype and expanded soluble Atxn3, highly sensitive immunoassays, so-called TR-FRET assays, were used. Thereby, we observed an increase in total soluble Atxn3 in both, WT/304Q and 304Q/304Q mice, at 3 months of age, which was reduced to WT/WT level in 18-month-old mice (Fig. 5E). Expanded Atxn3 was only measurable at both time points in WT/304Q and 304Q/304Q mice, but levels were reduced in 18-month-old mice when compared to 3-month-old mice (Fig. 5F). Importantly, the strongest reduction was found in homozygous animals where the highest amounts of aggregates were detected (Fig. 1B + C).

### Age-Dependent Gene Expression Changes Arise from Two Distinctive Perturbation Modes

**Fig. 6:**
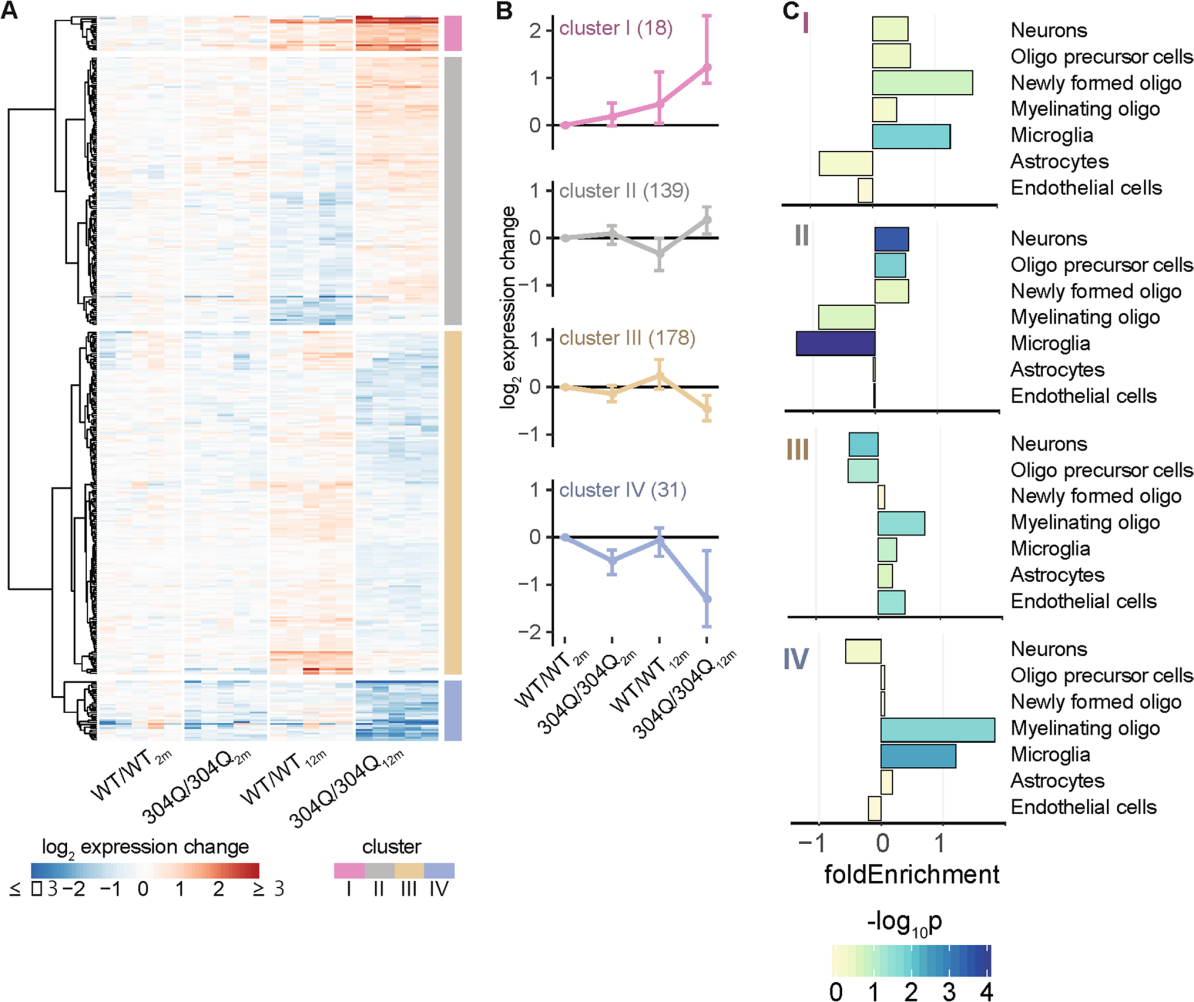
Modalities of age- and genotype-dependent gene expression perturbations. (**A**) Heatmap of hierarchically clustered expression levels (log_2_ expression change relative to WT/WT_2m_) across all experimental groups. Analyzed were the 366 DEGs found to be differentially expressed either in 2- or 12-month-old 304Q/304Q mice compared to their age-matched WT/WT littermates (see Fig. 5B for comparison). Based on their gene expression pattern, the 366 DEGs partition into four main clusters. (**B**) Subplots of each gene cluster shown as expression centroid (±SD). (**C**) Enrichment of DEGs for cerebellar cell types for each cluster based on cell type-specific reference data [33]. Bars show fold enrichments with color-coded p-values of two-sided Fisher’s exact tests

To better understand the age-dependent increase of expression perturbations in the cerebellum (Fig. 5A) of the 304Q/304Q model, all 366 DEGs (union of 2- and 12-month-old contrasts) were visualized across the experimental groups (Fig. 6A). Upon hierarchical clustering, these expression signatures partitioned into two primary classes. Class 1 comprises clusters I and IV, and class 2 comprises clusters II and III (Fig. 6B). Genes in class 1 showed an age-dependent aggravation of expression perturbations in 304Q/304Q animals that did not occur in WT animals.

Cell type-specific expression data from the Brain-Seq database [33], which reports the cerebellar expression level of genes across seven cell types, were used to determine enrichments among the 365 DEGs. This showed that especially in cluster I (astrocytes: Tpbg; microglia: Cd68, Cd74, Comp, Ier5, Igfbp4, Lyz2, Plcg2, St14, Trem2), but also in cluster IV (astrocytes: Hif3a, Igfbp5, Mt2, Nr4a2, Nr4a3; microglia: Car9, Ccdc63, Cd300a, Col27a1, Gcnt1, Kcnh3, Rasd2, Sult1a1), high expression changes were attributed to microglia and astrocytes (Fig. 6C). Therefore, we investigated if there was increased gliosis in the WT/304Q and 304Q/304Q mice by staining them for the microglia marker IbaI and the astrocyte marker Gfap (Online Resource SI S10). For the microglia, we observed a stronger activation in 3-month-old WT/304Q and 304Q/304Q compared to WT/WT, but no differences in 18-month-old mice (Online Resource SI S10A). For the astrocytes, we again observed a stronger activation in the young KI mice, but also in the 18-month-old ones (Online Resource SI S10A).

In class 2, the underlying genes underwent age-depended expression adaptations in WT/WT animals that, however, failed in KI mice or, for some genes, were even oppositely regulated. Similar modes of age-dependent gene expression changes have already been described in a BAC SNCA mouse model for Parkinson’s disease and might represent a common pattern for neurodegenerative diseases [44].

As the downregulated DEGs were enriched for structural constituent of myelin sheath (Fig. 5C), oligodendrocytes may play a role in the observed perturbations. To test this hypothesis, cell type-specific expression data from the Brain-Seq database [33], were again used to determine enrichments among the 365 DEGs. Indeed, downregulated genes, in particular the subset of DEGs in cluster IV, was enriched for genes attributed to myelinating oligodendrocytes (Fig. 6C).

### Shared Perturbances in Oligodendrocytes Between 304Q/304Q Mice and SCA3 Patients

**Fig. 7:**
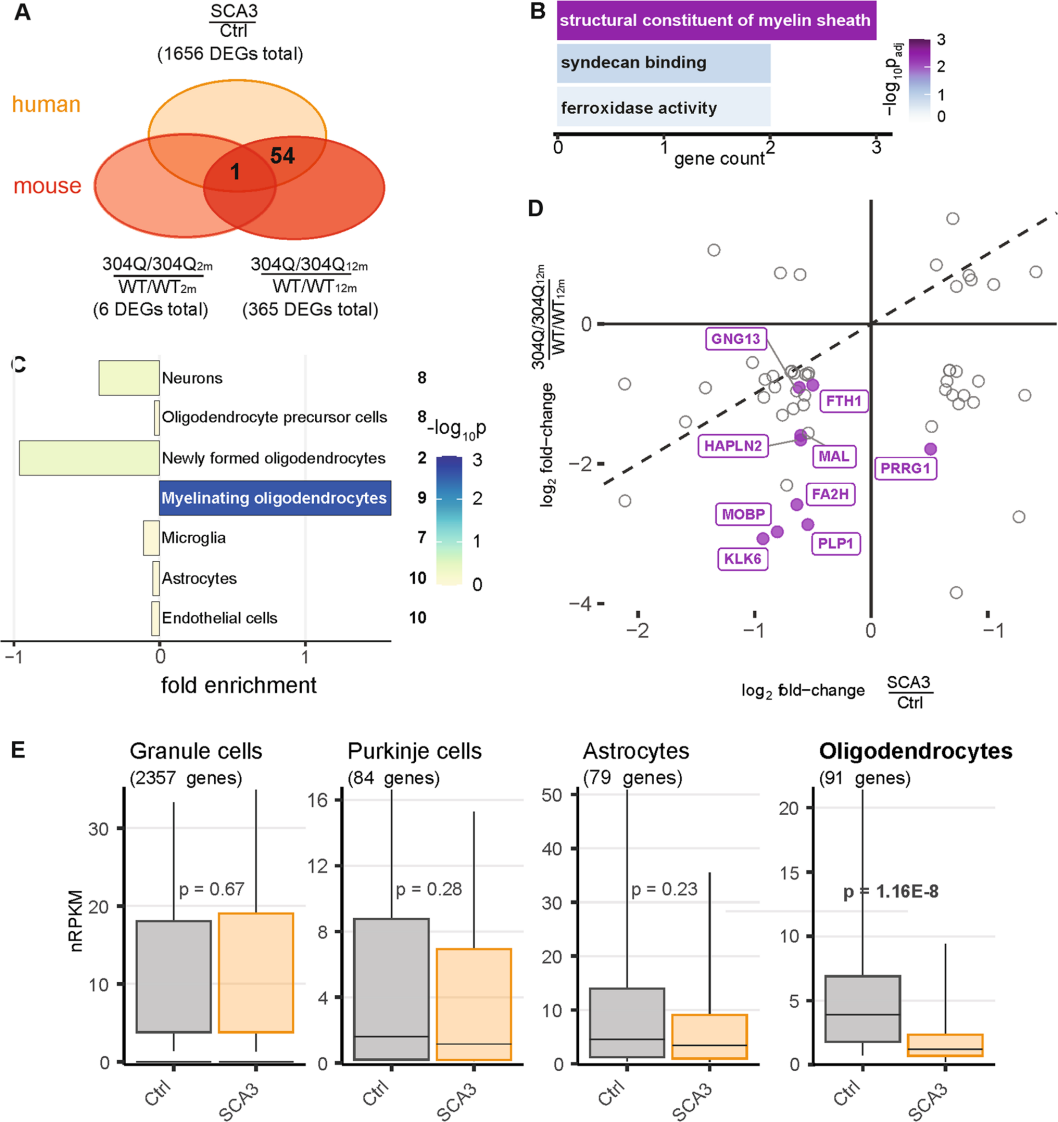
Common expression disturbances in KI mouse model and SCA3 patients point towards myelinating oligodendrocytes. (**A**) Venn diagram comparing DEGs in 2- and 12-month-old 304Q/304Q mice with orthologue DEGs found in the cerebellum of SCA3 patients. 55 genes were found to be differentially expressed in both mouse and human post-mortem cerebellum samples. (**B**) Enriched Gene Ontology terms (molecular function) for 55 DEGs shared between 304Q/304Q mice and human patients. (**C**) Enrichment of shared DEGs for cerebellar cell types according to cell type-specific reference data (Ref Brain-Seq [33]). Number of attributed genes indicated on the right. Bars show fold enrichments with color-coded p-values of two-sided Fisher’s exact tests. (**D**) Scatter plot of expression changes for 55 common DEGs identified in cerebellum of mouse and human. DEGs assigned to myelinating oligodendrocytes in purple. (**E**) Cell type-specific cerebellar gene expression in SCA3 patients and healthy controls. Boxplots show geometric mean and 10th, 25th, 75th, and 90th quantile of nRPKM values for all genes attributed to distinct types based on human reference data [34]. Number of genes per cell type in brackets. *P*-values of two-tailed Mann-Whitney *U* tests indicated for each cell type

To relate gene expression changes observed in mouse cerebellum to human, we investigated post-mortem cerebellar tissue of SCA3 patients and healthy controls using RNA-seq. Of 1656 DEGs identified in humans, 55 were also differentially regulated in the KI mouse model (Fig. 7A). These common DEGs were most significantly enriched for structural constituent of myelin sheath among molecular functions of Gene Ontology terms (Fig. 7B). To further investigate this re-occurring result for a potential role of oligodendrocytes, the 55 DEGs were classified by their cell-type-specific expression according to the Brain-Seq database [33]. The strongest enrichment for 55 attributable genes was found for myelinating oligodendrocytes (Fig. 7C). Eight out of nine genes (*FA2H, FTH1, GNG13, HPLN2, KLK6, MAL, MOBP, PLP1*, and *PRRG1*) underlying this term showed the same perturbation directionality when comparing 304Q/304Q KI mouse and SCA3 patient expression perturbations (Fig. 7D). Moreover, Ramani and colleagues showed that *mMyelin associated* *oligodendrocyte basic protein (Mobp)* was significantly downregulated in YAC84Q mice compared to YAC15Q and that *Kallikrein related peptidase 6 (Klk6)* and *m Myelin and lymphocyte protein* (Mal) were also affected in the aggregate-prone models (YAC84Q, KI-hom and dupKI-het) investigated by Ramani et al. [42] pointing to possible alterations in oligodendrocytes in these models.

The common downregulation of genes (3rd quadrant) attributed to oligodendrocytes prompted us to assess cell type composition effects. Using Brain-Seq reference data first for mice, the average expression of all genes attributed to myelinating oligodendrocytes indicated indeed a slightly reduced expression in 12-month-old 304Q/304Q mice (Online Resource SI 11A). Intriguingly, this reduction in oligodendrocytes was significant in SCA3 patients (Fig. 7E), when using a cell population-specific reference data for human cerebellum [34].

**Fig. 8:**
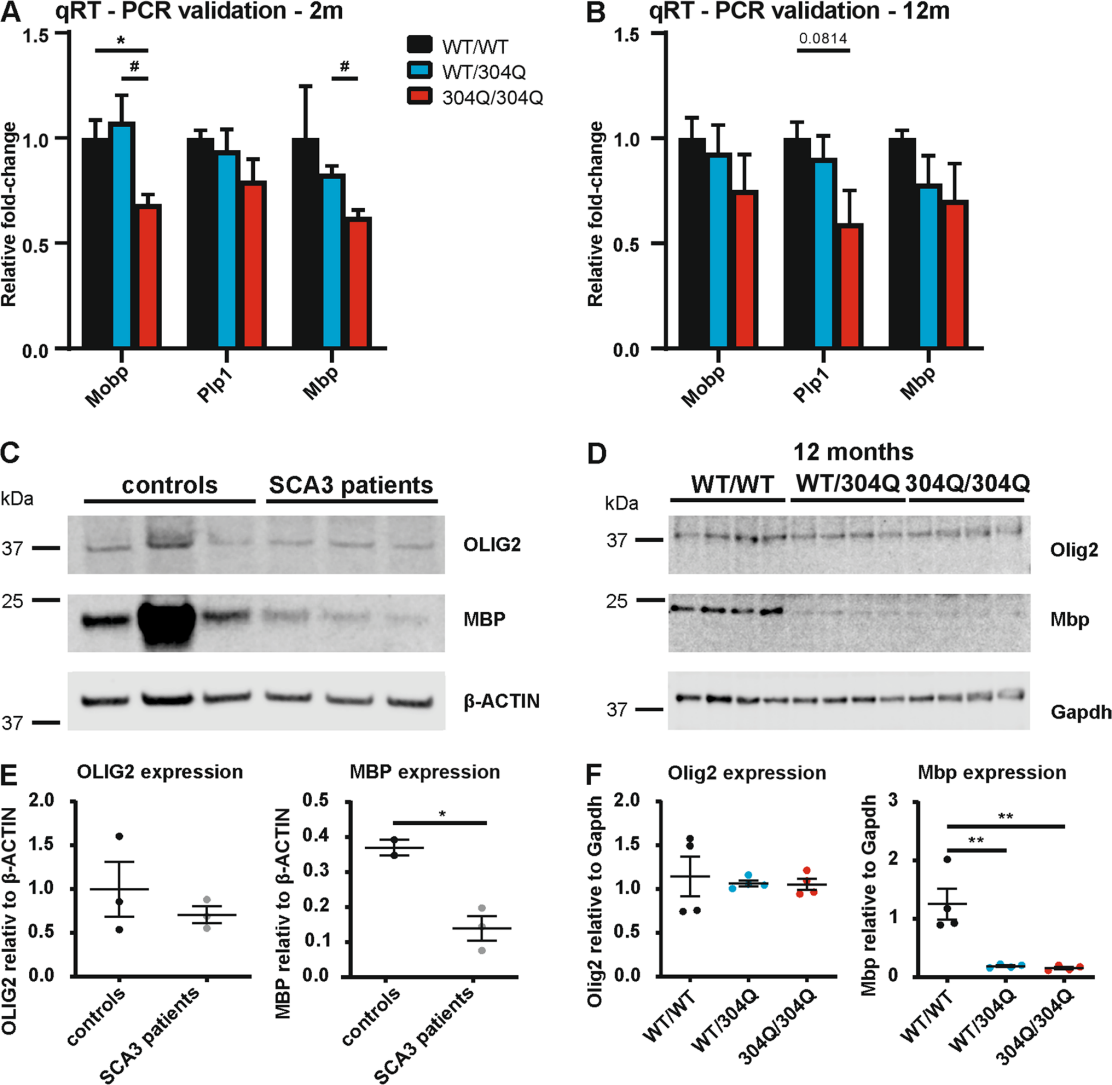
Expression of myelinating oligodendrocyte marker OLIG2 and MBP in human and mouse cerebellum. (**A**+**B**) qRT-PCR validation in RNA samples of 3-month-old (A) and 12-months-old (**B**) mice showed tendency of a downregulation of *Mobp, Plp1* and *Mbp* in 304Q/304Q KI mice compared to WT/WT littermates. (**C**+**E**) Western blot analysis of cerebellar lysates of OLIG2 and MBP in human cerebellar samples revealed a tendency of protein reduction in SCA3 patients for OLIG2 and a significant reduction for MBP compared to healthy controls. (D**+F**) In 12-month-old mice no difference of the Olig2 marker was observed, but a strong decrease of Mbp was detected in both, WT/304Q and 304Q/304Q mice compared to WT/WT mice. (**A**+**B**) n = 3, (C-F) n = 4, both sexes, two-tailed Student’s t-test. β-ACTIN or Gapdh shown as loading control

To further strengthen our hypothesis that oligodendrocytes are impaired in our SCA3 KI model we validated the gene expression of four oligodendrocyte markers by qRT-PCR (Fig. 8A + B). In 2-month-old mice *Mobp* expression was significantly reduced in 304Q/304Q compared to both WT/WT and WT/304Q littermates in cerebellar brain samples. *Myelin basic protein (Mbp)* expression was also significantly decreased in 304Q/304Q mice compared to WT/304Q mice. A tendency for a reduced gene expression was detected in 304Q/304Q compared to WT/WT mice for *Proteolipid protein 1 (Plp1)* (Fig. 8A). In 12-month-old mice we observed a tendency for a reduced gene expression for Mobp, Mbp and Plp1 without reaching significance (Fig. 8B).

Furthermore, in protein analyses for the oligodendrocyte transcription factor OLIG2 and MBP in cerebellar brain samples of SCA3 patients and healthy controls (Fig. 8C) we observed a tendency towards a reduction of OLIG2 and a significant reduction for MBP (Note: the second control was not considered for quantification to avoid overinterpretation of the data). A significant reduction for Mbp was observed in 12-month-old WT/304Q and 304Q/304Q mice compered to WT/WT littermates (Fig. 8D + F) however, we did not observe differences in the protein levels of Olig2 between the genotypes. In immunofluorescent staining for Olig2 in the DCN of the 304Q KI mice, we first observed an increase in the number of Olig2 positive cells in WT/304Q mice and 304Q/304Q mice compared to WT/WT mice (Online Resource SI 11B). Later, with 12-months of age, a reduction in the signal intensity was observed in the WT/304Q and 304Q/304Q mice compared to age-matched WT/WT littermates (Online Resource SI 11B). The same pattern was present in the pons (data not shown) another aggregate-prone brain area.

Taken together, these findings are in line with previous reports on SCA3 mouse models, which suggest an important role of oligodendrocytes [42, 43, 45, 46]. Further, our data are in line with the recent study by Costa and colleagues [47], who could show that MBP levels were reduced in homozygous YAC84Q mice and SCA3 patients. Taken together, this is strong evidence that oligodendrocyte impairment can be an important mechanism in SCA3 and can further be investigated with our new KI model.

## Discussion

**Fig. 9:**
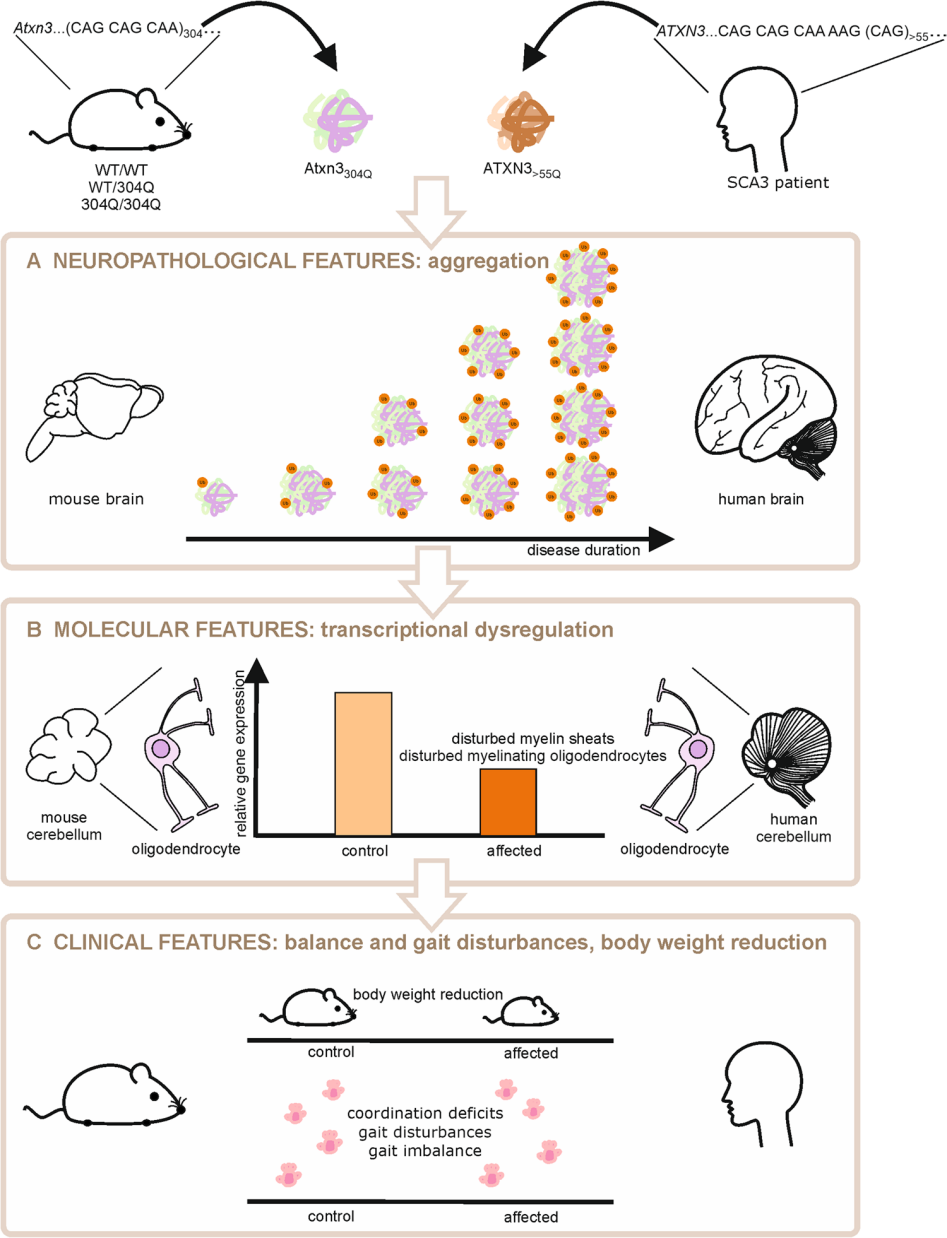
Summary of common findings of SCA3 features in human patients and our Atxn3 KI mice. The novel Ataxin-3 knock-in mouse model characterized in this study show neuropathological, molecular and phenotypical features resembling the clinical characteristics of human SCA3 patients. The disease phenotype developed by the knock-in mice is caused by the hyper-expanded trinucleotide repeat region of 304 CAACAGs either in one or both alleles for *Atxn3*. (**A**) The resulting disease protein harbors an abnormally extended polyQ stretch, forming Ub-positive Atxn3 aggregates in brain regions affected by SCA3. The aggregate formation, which is shared by knock-in mice and affected patients, is accompanied by further molecular aberrations (**B**), including transcriptional dysregulation. In both, knock-in mice and SCA3 patients, transcriptional alterations in oligodendrocytes are enriched for genes attributed to myelin sheaths and myelinating oligodendrocytes. (**C**) The phenotypical features developed by knock-in mice resembles the clinical manifestations of affected patients, encompassing body weight reduction, coordination deficits, gait disturbances and imbalance. Thus, the novel knock-in mouse model represents an appropriate disease model to investigate early-onset pathomechanisms, potential therapies and longitudinal biomarker alterations.

In this study, we generated and characterized a novel SCA3 KI mouse model that depicts the SCA3 clinical and neuropathological phenotype in patients in a more complete manner, than previously described SCA3 mouse models. With this new model, we could mimic neuropathological features, like the formation of aggregates and cell loss of Purkinje cells, but also reproduce a behavioral phenotype manifested by a reduction in body weight as well as balance and gait instability. Due to the interrupted repeat sequence CAACAGCAG, that was chosen to avoid intergenerational instability, we cannot investigate the effects triggered by RNA instability or repeat associated non-ATG (RAN) translation. However, the transcriptional changes in this model revealed disturbances in the myelin sheaths and in myelinating oligodendrocytes, changes that were shared with perturbations in the cerebellum of post-mortem SCA3 patients (Fig. 9). Former studies using our SCA3 KI model could also show an age- and aggregate- related increase of the blood biomarker NfL comparable to the NfL increase in SCA3 patients [11], a marker for neuronal loss in neurodegenerative disorders. Further, first non-allele-specific gene silencing studies, using miRNA were able to reduce the amount of expanded Atxn3 mRNA in these mice [24]. Therefore, this 304Q model is most suitable to investigate early-onset and longitudinal changes, making it an optimal candidate to study disease initiation, progression, and treatment.

It is known that KI models for SCA3 containing a CAG expansion within the range of repeat length expansions of human patients, display only a mild or no behavioral phenotype [22, 42]. The same is true for other polyQ diseases like SCA1 [48], SCA2 [49], or HD [50]. Especially for the latter it has been proven, that a hyper-expansion of the repeat is necessary to trigger a behavioral phenotype [51]. Moreover, the late-onset behavior phenotype in the hemizygous SCA3 YAC84Q model, which carry the full-length human *ATXN3* gene containing 84 CAG repeats and all regulatory elements [19], was often not reproducible in follow-up studies [43, 52]. This leads us to the assumption, that a hyper-expansion of the polyQ tract in the murine genome is necessary to trigger a phenotype comparable to human patients. Therefore, we decided to generate a new SCA3 KI mouse model with a hyper-expansion of more than 150 glutamines, that would be long enough to generate the desired phenotype. Further, we used an interrupted repeat, meaning a CAA instead of a CAG codon on every third position, both translated to glutamine on protein level. This bears the advantage to increase the stability of the resulting polyQ tract [53], which has not changed in our model since the first measurements of the fragment length in 2015. A disadvantage of the interrupted repeat, however, is that our mice could miss the feature of potential RNA toxicity, that is less prominent in interrupted repeats and can also contribute to disease progression [54, 55]. Further, it is not possible to assess the impact of repeat associated non-ATG (RAN) translation, which might also play a role in SCA3, but could be extenuated in our 304Q KI mice. However, RAN translation has so far only been proven in cell culture overexpression experiments with truncated forms of Atxn3 [56]. Therefore, the role of RAN translation in the SCA3 pathology must be further investigated, both in SCA3 patients and mouse models capable of capturing this specific feature of the disease.

For the characterization of our new KI model, we started with protein expression analysis. Our 304Q KI line revealed soluble Atxn3 protein expression throughout aging in the brain and several peripheral organs, including heart, lung, liver, kidney, spleen, and muscle tissue using different anti-Atxn3 antibodies to detect expanded Atxn3. These findings are mostly consistent with the protein expression pattern found by Switonski and colleagues [22] in their Ki97Q mouse line, which also demonstrated expanded Atxn3 expression in all tissues except of kidney. Next, we observed a significant increase of Atxn3- and Ub-positive aggregates in both WT/304Q and 304Q/304Q KI mice over time. This aggregate formation occurs already in early stages (first measured with 3 months of age), long before any behavioral changes get manifested. However, in our KI mouse line expressing only 97Q no aggregate formation was detected. When comparing aggregation in our WT/304Q mice and in the YAC84Q mice, we observed earlier Atxn3 aggregate formation in hemizygous YAC84Q mice but no increase over time, while strongest aggregation was found in the WT/304Q (304Q/304Q were not included in this experiment but showed even stronger aggregate formation than WT/304Q mice). Ubiquitination of the aggregates was only slightly measurable in 18-month-old hemizygous YAC84Q mice. Hence, the decision which mouse model is most suitable depends mainly on the desired readout of the experiments. In our new 304Q KI line, where we use the murine regulatory elements, a hyper-expansion is necessary to generate an early onset but progressive aggregate formation over time, accompanied by ubiquitination of these aggregates. The hemizygous YAC84Q mice on the other hand, which are using human regulatory elements, show earlier aggregate formation triggered by a more physiological repeat number, but fail to increase the amounts of aggregates over time as well as the in human patients described ubiquitination.

In the next step, we observed that more soluble Atxn3 protein was expressed in the 304Q KI mice compared to WT/WT mice with 3 months of age, but that this level was reduced in 18-month-old mice, indicating its inclusion into aggregates. The localization of these aggregates is ubiquitous in the brain, but especially prominent in the hippocampus, pons and DCN. All these areas are known to be vulnerable in SCA3 patients [9, 10]. The localization of the aggregates is comparable to the findings of Ramani and colleagues [20, 21] in their dupKi mouse. Another neuropathological variation we observed, was a reduction in the numbers of intact Purkinje cells. Here, we could already detect a reduction of intact PCs in 3-month-old 304Q/304Q KI mice persistent with aging. In the heterozygous WT/304Q this reduction became significant with 12 months of age, comparable to the results observed for the hemizygous YAC84Q mice. Changes in Purkinje cell pattern and atrophy have been reported before, both for SCA3 mouse models [22, 57] and in histopathological examinations of SCA3 patients [37]. Whether or not there is a connection between neuronal cell loss, cellular dysregulation, cerebellar function, and a motor phenotype is not completely clear yet. While Shakkottai and colleagues [58] reported of changes in Purkinje cell firing concurring with behavioral deficits in YAC84Q mice even before observable neurodegeneration was detected, Costa Mdo [59] described behavioral deficits in the same mouse model but could not detect changes in Purkinje cell count. Toonen and colleagues [43] did not observe behavioral differences at the same time point. Therefore, the relation between Purkinje cell loss and a motor phenotype needs further investigation comparing several SCA3 models to human post-mortem brain analyses. Taken together our findings, that despite we see only few aggregates in the cerebellum with 3 months on the one hand, but a reduced number of intact PCs at the other hand, we assume that PCs are early affected in SCA3, but are not main carriers of Atxn3 aggregates. Scherzend and colleagues [37] speculate in SCA2 and SCA3 that the pathology of PCs could be the initial pathological change. However, if this is a neurodegenerative rather than a developmental effect remains unclear and needs further investigation.

These neuropathological features described above also have an impact on the phenotypical development of the KI mice. In male mice, we observed early reduction of body weight in heterozygous and homozygous animals, whereas the WT/304Q mice became later significantly different compared to their WT/WT littermates and were not as severely affected as the 304Q/304Q mice. The hemizygous male YAC84Q showed a similar reduction of the body weight as the 304Q/304Q mice for over a year and stayed on steady level, while a further drop of the body weight was observed for the 304Q/304Q mice. Female WT/304Q KI mice were not affected by body weight reduction, and 304Q/304Q females were significantly reduced compared to WT/WT littermates, but not nearly as early and strong as the males. The hemizygous female YAC84Q mice again behaved similar than the female 304Q/304Q mice. Body weight reduction has been shown earlier for the hemizygous YAC84Q mice [19] and is consistent with SCA3 patients, where a negative correlation between CAG repeat length and body weight has been proven [39, 40]. The discrepancy we noticed between male and female mice has also been reported for KI and transgenic mice of other polyQ diseases. In Huntington’s research, for example, male transgenic mice and rats, as well as the CAG140 KI mice, are more severely affected by body weight reduction than their female littermates [60, 61]. These differences in body weight affect also later experiments. Therefore, we observed physiological changes like size reduction and hunchback formation mainly in male mice. During RotaRod experiments, homozygous male mice seem to benefit from the reduced body weight at intermediate and become comparable to WT/WT littermates in later time points. However, in female mice, which are less affected by changes in body weight, we observed a tendency to perform worse in this motor task from the beginning and being significantly worse at late stages. However, in the statistical RotaRod analyses we took the co-variable “body weight” into account. Regardless, these sex discrepancies of heterozygous and homozygous 304Q KI mice as well as hemizygous YAC84Q mice must be kept in mind for further studies. Nevertheless, we do not recommend working with male or female mice only, when dealing with a disease in which male and female patients are impaired equally. Other behavioral experimental setups, like the Catwalk experiment, were seemingly unhindered by body weight changes and therefore we did not observe significant sex differences. Here, we detected increased step cycles in front and hind paws, decreased print area in front and hind paws, and increased base of support for the hind paws. Results were always significant in 304Q/304Q mice and for the base of support and the step cycle in the hind paws also for WT/304Q mice. These findings show that the detected changes in phenotypical parameters of our novel KI model are comparable to the ataxic phenotype in human patients, where differences in posturing for better balance are also described [4].

These measurable and quantifiable parameters, like aggregate formation, weight reduction, and motor phenotype, render these 304Q KI mice as an optimal model system for therapy studies or biomarker validations. Promising results with this exact model have already been achieved in first studies with microRNA therapy and longitudinal biomarker evaluations. Martier and colleagues [24] could recently show that the amount of expanded Atxn3 mRNA in our 304Q KI mice can be reduced by delivering microRNAs in an adeno-associated-vector system. Moreover, Wilke and colleagues were using this model to investigate the progression of NfL and pNfH as potential easily accessible serum biomarkers in a longitudinal study with mice in the age ranging from 2 to 24 months. They could prove the increase of NfL and pNfH already in 6-month-old mice, long before any symptoms developed and were able to correlate their findings with the respective protein levels in human patients [11]. Here, our model has a major advantage over other SCA3 mouse models since NfL is a potential primary or secondary readout parameter in future therapeutic trials and this KI model represents human pathology and neurodegeneration. However, we admit that our SCA3 KI model is not suitable for certain applications like gene therapy approaches, targeting for example the single nucleotide polymorphism SNP rs12895357, directly C-terminal of the polyQ repeat [62]. For these kind of studies expression of the human *Atxn3* gene and its regulatory elements is necessary and therefore further humanized mouse model are highly needed.

Another advantage of our SCA3 KI model is, that, it was possible to track first abnormalities in walking behavior by DeepLabCut software based on deep learning networks, already in 9-month-old homozygous KI mice, a time point before motor deficits were detectable with other behavioral experiments [41].These findings are in line with the changes observed in SCA3 patients, where gait biomarkers can be linked to SCA3 progression [63]. These findings support further the potential of our new KI mouse line for biomarker or treatment studies.

Potential biomarkers could also arise from high powered transcriptomic studies. In line with two other SCA3 rodent models, Interleukin-33 (Il33) is persistently downregulated in cerebellar samples of the 304Q/304Q KI mice at both investigated time points (2 and 12-months of age). In YAC84Q mice the Il33 transcript is also downregulated in brainstem samples of 6-month-old SCA3 mice [42] and whole brain samples of 17.5-month-old mice [43]. In addition to this, Il33 shows highest expression levels in the central nervous system, being constitutively expressed in astrocytes, microglia, and oligodendrocytes [64]. Moreover, a deficiency for Il33 is associated with neurodegeneration and impaired repair of neurons in aged mice accompanied by memory loss and cognitive impairment, resembling an Alzheimer’s disease-like phenotype, including tau abnormality [65].

Another candidate gene as a potential transcriptional biomarker is synapse differentiation inducing 1 like (Syndig1l), also known as Capucin. This gene was not only upregulated in our 2- and 12-month-old KI mice, but has also been shown to be differentially expressed in rodent models for HD [66], although its exact function in health and disease remains to be investigated.

Not only Il33 is associated with oligodendrocytes, but also our cell-type-specific data analysis point at disturbances in myelinating oligodendrocytes and myelinating sheaths. Importantly, these gene expression changes of Mobp, Mal, and Plp1 were shared between our SCA3 KI mice and human post-mortem cerebellar SCA3 patient tissue. A high number of downregulated genes in both, the KI mice and humans were enriched for this cell type. These findings are in line with the transcriptomic studies of Ramani [42] and Toonen [43] in the YAC84Q mice, with oligodendrocytes appearing mainly affected by transcriptional changes. Ramani and colleagues further showed that Atxn3 is present in the cell nuclei of oligodendrocytes and that gene expression of oligodendrocyte markers like Mobp, Mal and Klk6 were affected in the YAC84Q mice and their dupKI model. In a later study McLoughlin and colleagues [67] demonstrated, that oligodendrocytes can be directly targeted by antisense oligonucleotides, reversing changes in gene expression, e.g., the upregulation of oligodendrocyte specific genes. In SCA3 patients, the aggregation of ATXN3 in oligodendrocytes has yet not been investigated, but ATXN3 aggregation in Schwann cells, the myelinating cells of the peripheral nervous system, of SCA3 patients has been proven [68]. Moreover, Costa [47] presented in a recent study the in vivo molecular signature of SCA3. They showed that in homozygous YAC84Q mice and heterozygous MJD135 (135Q) mice neuronal cell loss and dysfunction was indicated by lower N-acetylaspartate (NAA) levels and disturbances in the phospholipid membrane metabolism and demyelination was indicated by lower myto-inositol and total choline levels. In the same study they showed that these mice and SCA3 patients present with reduced levels of cerebellar NfL and MBP, which is replicated also in our new SCA3 KI model. This reflects the progressive loss of gray and white matter in the brain of SCA3 patients, as shown for example in MRI studies by Kang and coworkers [69]. White matter changes have also been reported in other polyQ diseases. Jin and colleagues [70] were able to show early myelination defects in a Q250 mouse model of HD, possibly caused by altered oligodendrocyte differentiation. Further, the overexpression of htt in oligodendrocytes provokes behavioral deficits and increased demyelination in mice [71]. Up until now, white matter changes have been assumed to arise secondary after neuronal loss occurred. However, this study together with others supports the hypothesis that the expression of expanded Atxn3 in oligodendrocytes might impair proper myelination in parallel to or even proceeding neuronal cell damage. This could be common mechanism leading to neurodegenerative diseases, not only in polyQ diseases like SCA3 and HD, but for example also for Parkinson disease [72].

Taken together, we present a novel SCA3 KI mouse model that represents the human patient phenotype on a neuropathological, behavioral, and transcriptomic level in a more complete manner than any existing SCA3 mouse and is, therefore, an ideal model system for further studies, including therapy and biomarker investigations.

## Supplementary Information

ESM 1 (PDF 2435 kb)

## Data Availability

KI raw sequencing files are available through GEO (https://www.ncbi.nlm.nih.gov/geo/query/acc.cgi) under accession number: GSE145613 and after consent of the corresponding author. Human RNA-seq data set has been deposited at the European Genome-phenome Archive (EGA) (https://www.ebi.ac.uk/ega/home), which is hosted by the EBI and the CRG, under the accession number: EGAS00001004241.

## Abbreviations

ABC: avidin-biotin-complex
Atxn3: Ataxin-3
BMI: body mass index
BOS: base of support
DCN: deep cerebellar nuclei
DEGs: differentially expressed genes
DSB: double-strand-break
GL: granular layer
GO: gene ontology
HD: Huntington’s disease
HR: homologous recombination
Il20rb: interleukin-20 receptor beta
Il33: Interleukin-33
Klk6: Kallikrein Related Peptidase 6
Mal: Myelin and lymphocyte protein
Mbp: Myelin basic protein
MJD: Machado-Joseph disease
ML: molecular layer
Mobp: Myelin associated oligodendrocyte basic protein
NAA: N-acety aspartate
NfL: neurofilament light chain
NGS: normal goat serum
nRPKMs: normalized Reads Per Kilobase per Million total reads
PA: print area
PCs: Purkinje Cells
PCR: polymerase chain reaction
PFA: paraformaldehyde
Plp1: Proteolipid protein 1
pNfH: phosphorylated neurofilament heavy chain
Q: glutamine
KI: knock-in
RNA-seq: RNA sequencing
SC: step cycle
SCA3: Spinocerebellar ataxia type 3
Syndig1l: Synapse differentiation-inducing gene protein 1-like, tb: terbium cryptate
TR-FRET: Time-Resolved Fluorescence Resonance Energy Transfer
Ub: ubiquitin
WT: wildtype/non-expanded
Zn: Zinc
