# Supplementary material for: A Novel SCA3 Knock-in Mouse Model Mimics the Human SCA3 Disease Phenotype Including Neuropathological, Behavioral, and Transcriptional Abnormalities Especially in Oligodendrocytes
Source: Mol Neurobiol. 2021 Oct 30;59(1):495–522. doi: 10.1007/s12035-021-02610-8 (PMC8786755; doi:10.1007/s12035-021-02610-8)
Supplement: Supplementary file 1 — (PDF 2435 kb) [file 12035_2021_2610_MOESM1_ESM.pdf]

Supplement Information (SI)

A novel SCA3 knock-in mouse model mimics the human SCA3 disease phenotype including neuropathological, behavioral, and transcriptional abnormalities especially in oligodendrocytes

Eva Haas[1,2], Rana D. Incebacak[1,2], Thomas Hentrich[1,2], Chrisovalantou Huridou[1,2], Thorsten Schmidt[1,2], Nicolas Casadei[1,2,3], Yacine Maringer[1,2], Carola Bahl[1,2], Frank Zimmermann[4], James D. Mills[5], Eleonora Aronica[5], Olaf Riess[1,2,3], Julia M. Schulze-Hentrich[1,2], Jeannette Hübener-Schmid[1,2]

MOLECULAR NEUROBIOLOGY

Correspondence: Dr. Jeannette Hübener-Schmid, Institute of Medical Genetics and Applied Genomics, University of Tübingen, Tübingen, Germany,

E-Mail: Jeannette.Huebener@med.uni-tuebingen.de

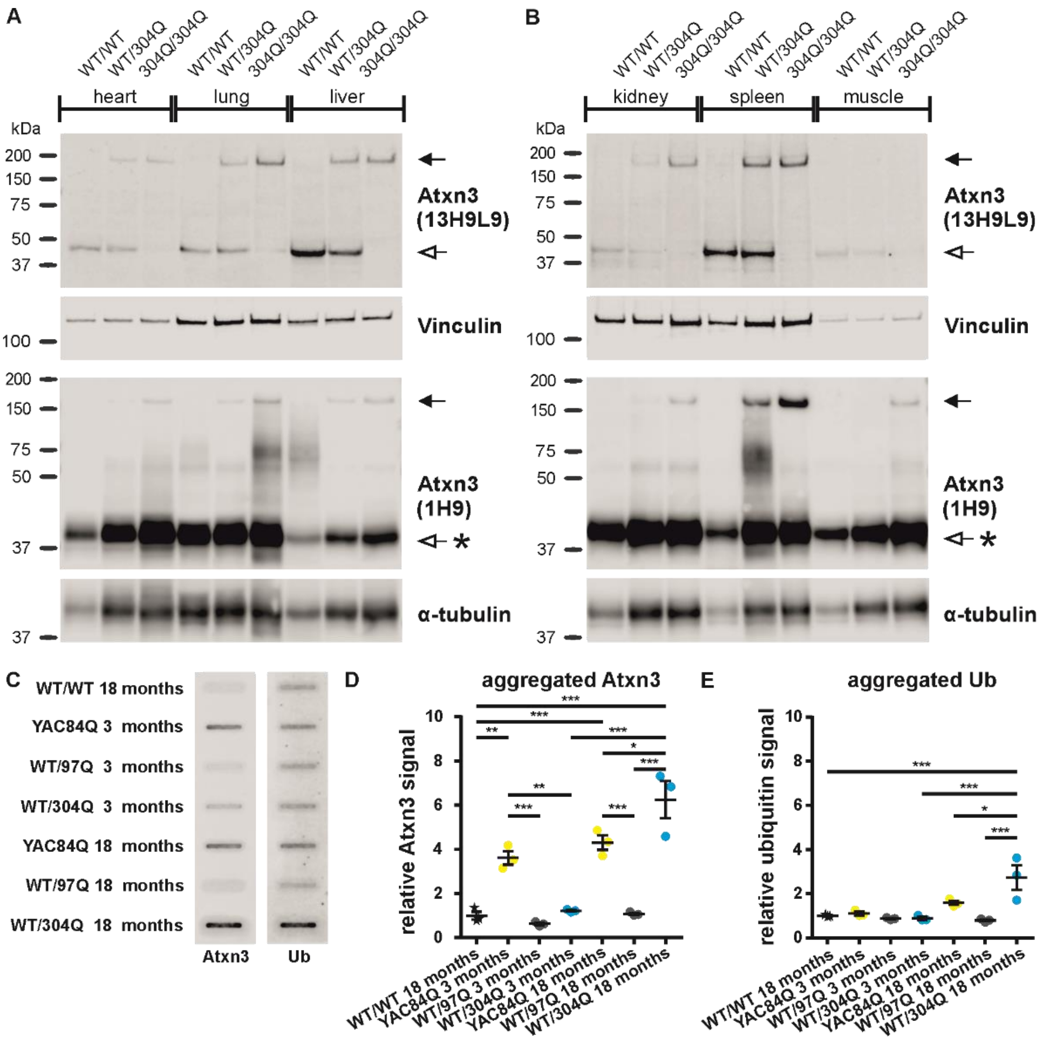

Online Resource SI1 (.tif) Protein expression of expanded Atxn3 in peripheral organs. (A + B) Expression of non-expanded and polyQ-expanded Atxn3 in KI line 304Q is detectable in heart, lung, liver (A), kidney, spleen and muscle (B). In muscle tissue detection was only possible with mouse-anti-Atxn3 (1H9) antibody not with rabbit-anti-Atxn3 (13H9L9) antibody. mouse-anti-Atxn3 (1H9) antibody covers signal of non-expanded Atxn3 by recognizing murine IgG light chain (indicated by *) (C-E) Formation of aggregated Atxn3 and Ub was measured by filter retardation assay (C) in whole brain samples of WT/WT, WT/97Q, YAC84Q and WT/304Q mice with 3 and 18 months of age. (C+E) First Atxn3-positive aggregates appeared in 3-month-old YAC84Q mice with no further increase in 18-month-old mice. Highest amounts were measured in 18-month-old WT/304Q mice. WT/97Q and WT/WT mice did not form any aggregates. (C+E) Ub-positive aggregates were only detectable in 18-month-old WT/304Q and none of the other genotypes. (A+B) n = 1 per genotype including both sexes (C-E) n = 3 per genotype, mixed sexes; filled arrow = polyQ-expanded Atxn3, unfilled arrow = non-expanded Atxn3; * IgG artefact. Vinculin or α-tubulin were used as loading control; yellow= YAC84Q, grey = WT/97Q, cyan = WT/304Q

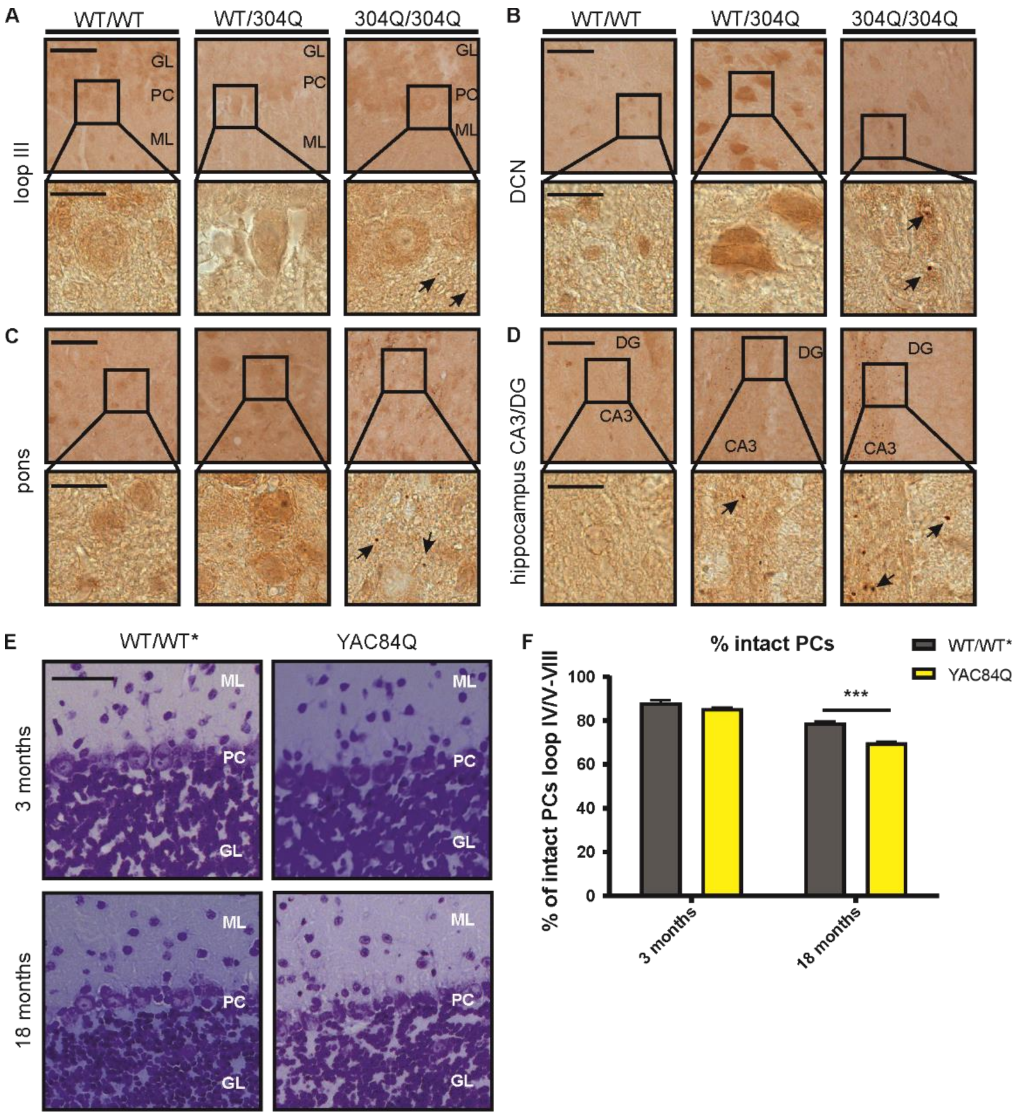

Online Resource SI2 (.tif) Beginning of aggregation of expanded Atxn3 in 3-month-old SCA3 KI mouse brain. Immunohistochemical (IHC) staining using Atxn3-specific antibody (clone 1H9) showed aggregate formation in WT/304Q and 304Q/304Q mice. IHC of 3-month-old mouse brains revealed increased diffuse nuclear staining in WT/304Q in the molecular layer of loop III (A), the DCN (B) and the pons (C) and beginning of aggregate formation in the hippocampus (D). In 304Q/304Q mice, first aggregates in the molecular layer of loop III, DCN, pons, and hippocampus (A-D) can be observed at 3 months of age. (D-F) The number of intact PCs in the cerebellar loops IV/V to VIII were determined with cresyl violet staining in sections of 3- and 18-month-old YAC84Q mice (E) and revealed a significant reduction of intact PCs with 18 months of age in YAC84Q mice compared to WT/WT*. (A-E) Scale bar = 50 µm, inset scale bar = 20 µm. Aggregated Atxn3 are indicated by arrows. n=3; GL = granular layer, PC = Purkinje cells, ML = molecular layer; DG = dentate gyrus, DCN = deep cerebellar nuclei

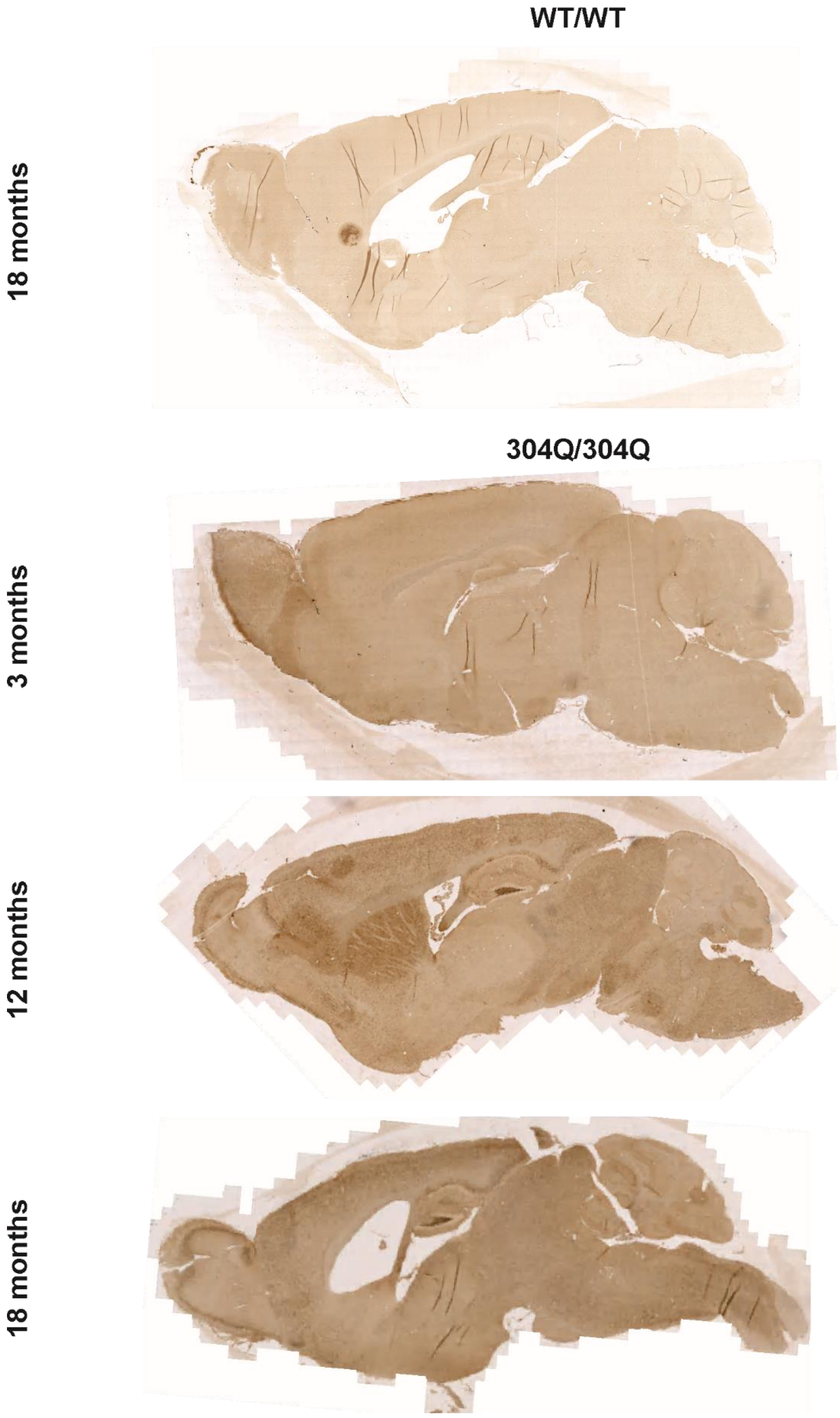

Online Resource SI3 (.tif) Overview of 304Q/304Q sagittal sections with 3, 12 and 18 months of age compared to 18 months old WT/WT. Aggregate formation increased over time in 304Q/304Q mice. The increase is detectable especially in the olfactory bulb, cerebral cortex, hippocampus, cerebellum, and hindbrain by darkening of the tissue as the number of Atxn3 aggregates increases. n = 3, both sexes

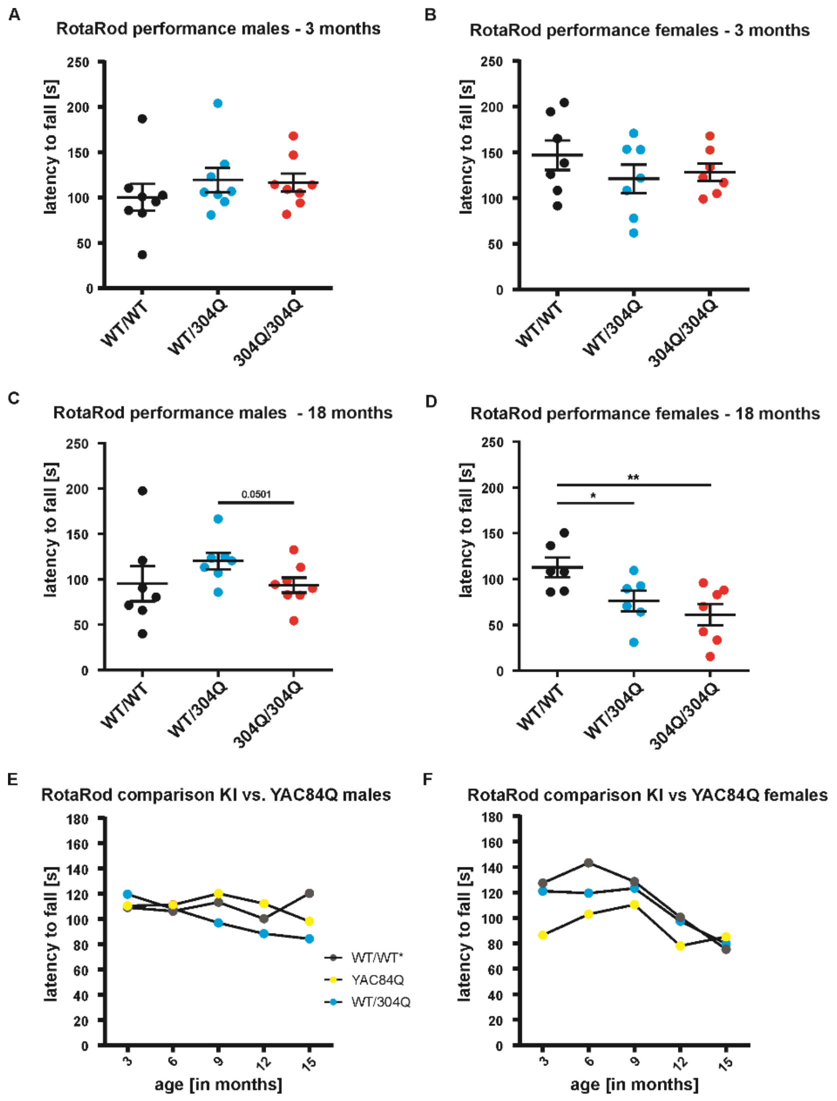

Online Resource SI4 (.tif) Coordination on RotaRod in male and female SCA3 KI mice and comparison to the YAC84Q model. At 3 months of age, no significant differences in RotaRod performance were observed in either male (A) or female (B) SCA3 KI mice. (C) At 18 months of age, a tendency in homozygous 304Q/304Q male mice was observed to perform worse than their heterozygous littermates. (D) In 18-month-old WT/304Q and 304Q/304Q female mice coordination was significantly decreased compared to WT/WT littermates. (E) Male YAC84Q mice do not differ in their RotaRod performance compared to their WT/WT* controls. In comparison, WT/304Q mice performed worse, when compared to these mice (independent experiments). (F) Female YAC84Q mice performed worse than their WT/WT* littermates on the RotaRod and worse than the female WT/304Q mice from the other experimental setup (independent experiments). (A– D) n = 6 - 8 mice per genotype including both sexes, two-tailed Student's t-test with Welsh correction, (E-F) male n = 6 - 10, females n = 4 - 9; Shapiro-Wilk test and two-way ANOVA adjusted for sex and body weight as covariable using IBM SPSS Statistics version 27; black = WT/WT, cyan = WT/304Q, red = 304Q/304Q, grey = WT/WT* (control to YAC84Q), yellow = YAC84Q

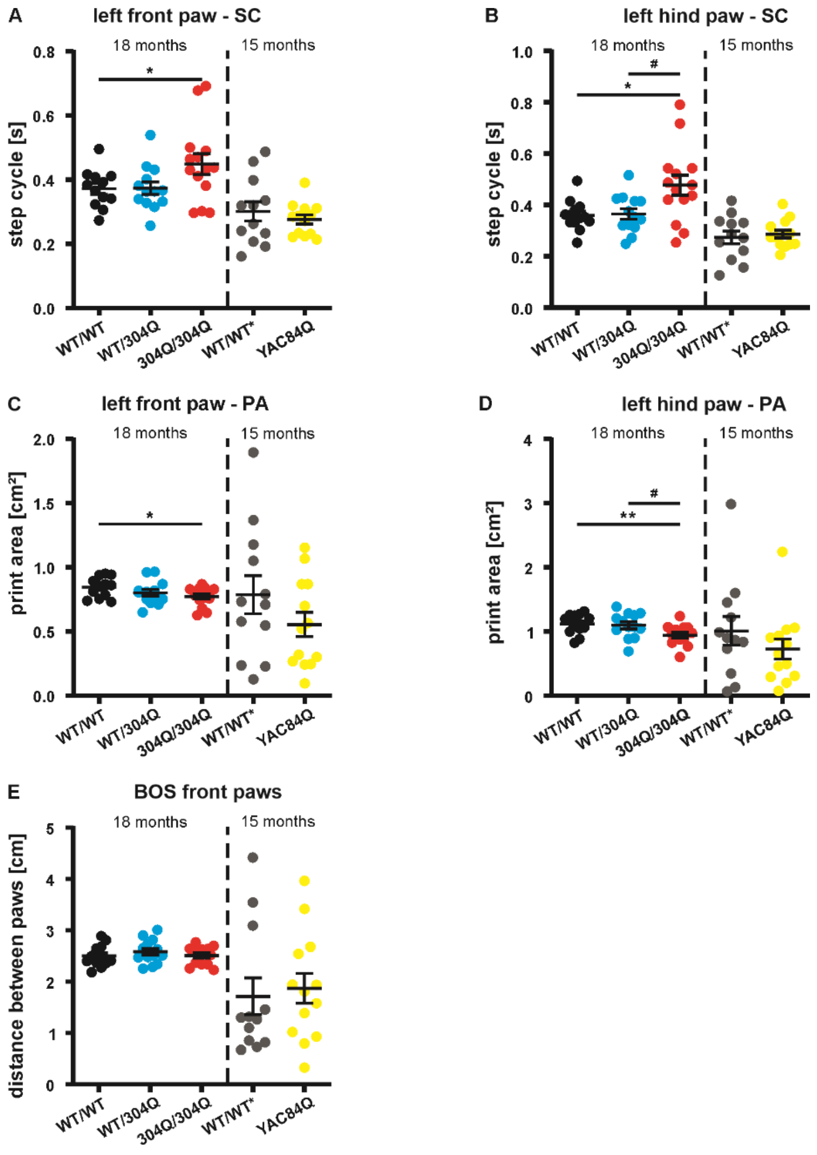

Online Resource SI5 (.tif) Altered paw positioning (left side) in 18-month-old WT/304Q and 304Q/304Q KI mice compared to 15-month-old YAC84Q mice. (A-B) Step cycle (SC) was increased for 304Q/304Q in the left front paw compared to WT/WT littermates (A) and to WT/WT and WT/304Q littermates for the left hind paw (B). (C-D) Print area (PA) of the left front (C), and hind paw (D) was significantly reduced in 304Q/304Q mice. For the hind paw, this reduction was also significantly different to WT/304Q mice. (E) Base of support (BOS) of the front paws was not altered. No differences were observed between YAC84Q mice and their WT/WT* controls. n = 12-16 mice per genotype both sexes, two-tailed Student's t-test with Welsh correction, *or # $p < 0.05$, ** $p < 0.01$, *** or ### $p < 0.001$, * comparison WT/WT to KI lines, # comparison WT/304Q to 304Q/304Q, black = WT/WT, cyan = WT/304Q, red = 304Q/304Q, grey = WT/WT* (control to YAC84Q), yellow = YAC84Q

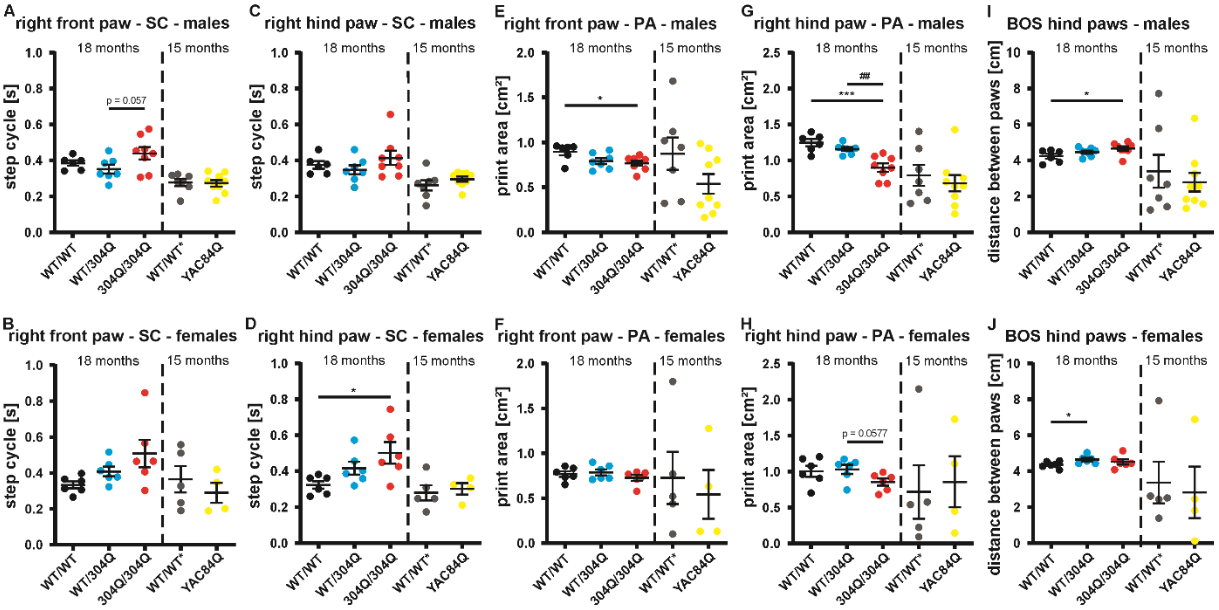

Online Resource SI6 (.tif) Altered paw positioning of the right paw in old WT/304Q and 304Q/304Q KI mice separated by sex. (A-J) Gait analyses separated by sex revealed less significant impairment for WT/304Q and 304Q/304Q mice compared to their WT/WT littermates as in the pooled cohort. (A+C) In male mice, step cycle (SC) was not increased in either right front (A) or right hind paw (C). For the front paw (A) a tendency for an increased SC was detectable between WT/304Q and 304Q/304Q KI mice. (B+D) In female mice, SC was not increased in the right front (B), but in the right hind paw (D) SC increased significantly in 304Q/304Q mice compared to WT/WT littermate. (E+G) Print area (PA) of the right front (E) and right hind paw (G) was significantly reduced in 304Q/304Q male mice compared to WT/WT littermates. For the hind paw, this reduction was also significantly different to WT/304Q littermates (G). (F+H) In female mice, no significant reduction in PA of the right front (F) and right hind paw (H) were detected. For the hind paw, a tendency for a reduction was observed in 304Q/304Q mice compared to their heterozygous littermates (H). (I-J) Base of support (BOS) of the hind paws was significantly increased in 304Q/304Q compared to WT/WT male littermates (I) and in WT/304Q female mice compared to WT/WT littermates (J). No differences were observed between YAC84Q mice and their WT/WT* controls. n = 6-8 mice per genotype and sex (KI lines), n = 4-9 mice per genotype and sex (YAC84Q line), two-tailed Student's t-test with Welsh correction, * $p < 0.05$, ## $p < 0.01$ *** $p < 0.001$, * comparison WT/WT to KI lines, # comparison WT/304Q to 304Q/304Q, black = WT/WT, cyan = WT/304Q, red = 304Q/304Q, grey = WT/WT* (control to YAC84Q), yellow = YAC84Q

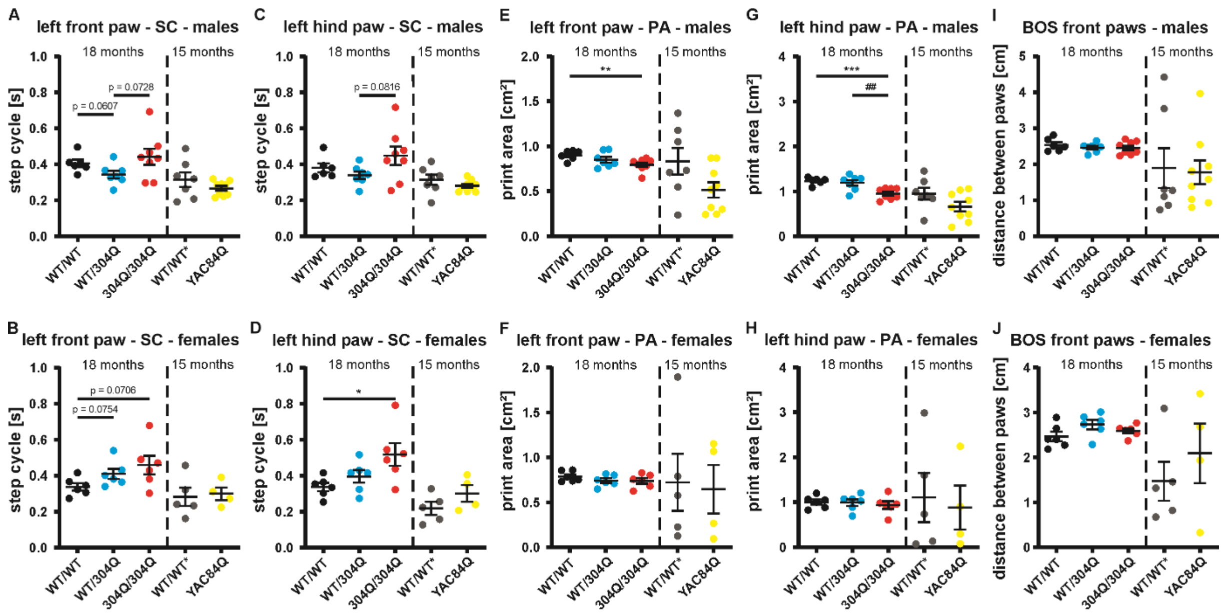

Online Resource SI7 (.tif) Altered paw positioning of the left paw in old WT/304Q and 304Q/304Q KI mice separated by sex. (A-J) Gait analyses separated by sex revealed less significant impairment for WT/304Q and 304Q/304Q mice compared to their WT/WT littermates as in the pooled cohort. (A+C) In male mice step cycle (SC) was not significantly increased in either left front (A) or left hind paw (C). For the front paw (A) a tendency for a decreased SC was detectable between WT/304Q and WT/WT mice. 304Q/304Q showed a tendency for increased SC compared to WT/304Q in the front (A) and hind paw (C). (B+D) Female WT/304Q and 304Q/304Q mice showed tendencies of an increased SC in the left front paw compared to WT/WT (B), and a significant increase in 304Q/304Q mice in the left hind paw compared to WT/WT (D). (E+G) Print area (PA) of the left front (E) and left hind paw (G) was significantly reduced in 304Q/304Q male mice compared to WT/WT littermates. For the hind paw this reduction was also significantly different to WT/304Q littermates (G). (F+H) In female mice no significant reduction in PA of the left front (F) and left hind paw (H) were detected. (I-J) Base of support (BOS) of the front paws was comparable between all groups. No differences were observed between YAC84Q mice and their WT/WT* controls. n = 6-8 mice per genotype and sex (KI lines), n = 4-9 mice per genotype and sex (YAC84Q line), two-tailed Student's t-test with Welsh correction, * $p < 0.05$, ## $p < 0.01$ *** $p < 0.001$, * comparison WT/WT to KI lines, # comparison WT/304Q to 304Q/304Q, black = WT/WT, cyan = WT/304Q, red = 304Q/304Q, grey = WT/WT* (control to YAC84Q), yellow = YAC84Q

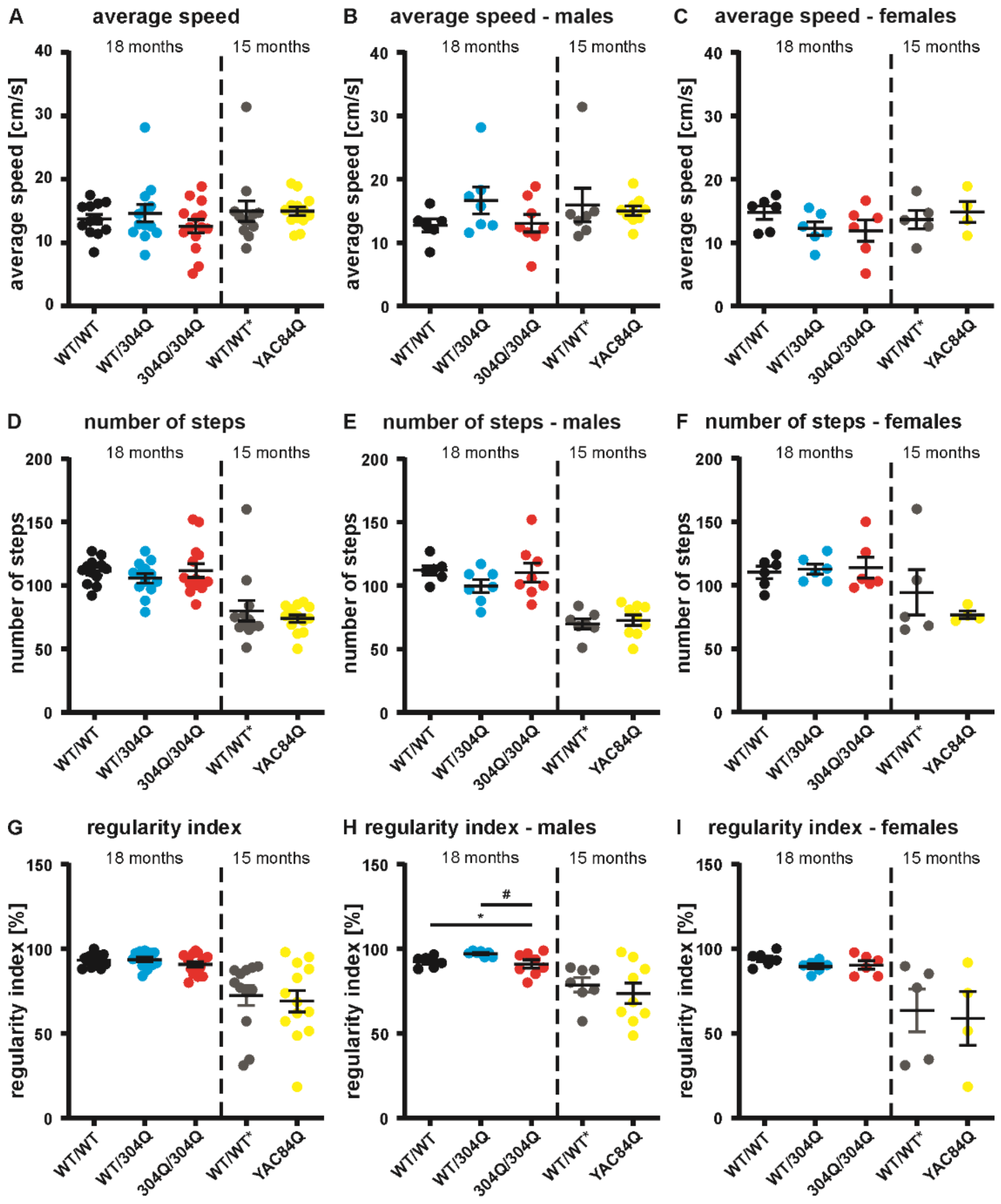

Online Resource SI8 (.tif) Additional parameters investigated by CatWalk gait analysis system. Average speed (A-C), number of steps (D-F), and the regularity index (G-I) were investigated. In male mice the regularity index was increased in WT/304Q mice compared to WT/WT and reduced in 304Q/304Q mice compared to WT/304Q mice. No differences between genotypes were observed in all other investigated parameters. No differences were observed between YAC84Q mice and their WT/WT* controls. n = 12-16 per genotype including both sexes, n = 6-8 mice per genotype and sex (KI lines), n = 4-9 mice per genotype and sex (YAC84Q line), for separated sexes, two-tailed Student's t-test with Welsh correction, * $p < 0.05$, ## $p < 0.01$ *** $p < 0.001$, * comparison WT/WT to KI lines, # comparison WT/304Q to 304Q/304Q, black = WT/WT, cyan = WT/304Q, red = 304Q/304Q, grey = WT/WT* (control to YAC84Q), yellow = YAC84Q

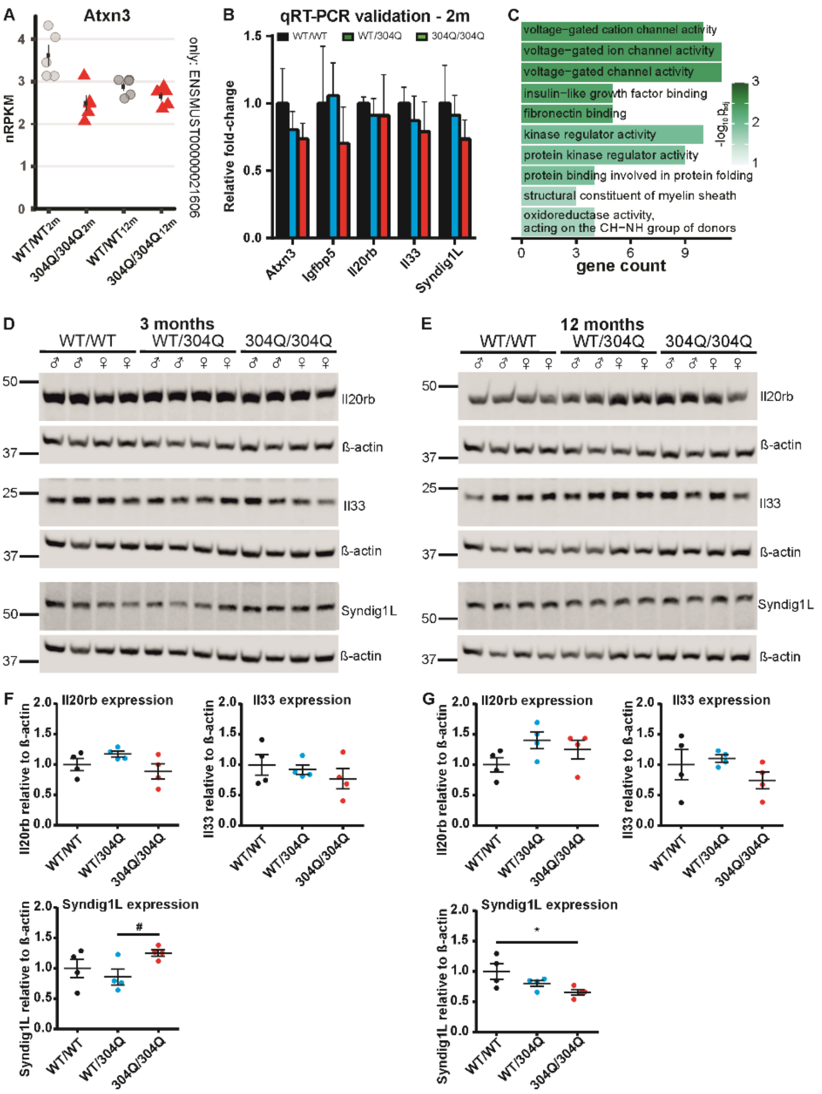

Online Resource SI9 (.tif) Altered gene and protein expression in SCA3 KI mice. (A) Cerebellar RNA expression for total or full-length *Atxn3* analyzed by RNA-seq showed increased gene expression in 2-month-old WT/WT mice and lower expression in all other groups. Expression is shown as normalized reads per kilobase per million total reads (nRPKM). (B) qRT-PCR validation in cerebellar RNA samples of 2-month-old mice did not confirm significant down- and upregulation of candidate genes in 304Q/304Q mice compared to WT/WT. For all genes, a tendency towards a downregulation could be observed, but never reached significance. (C) Overrepresented molecular functions among 365 DEGs found in 12-month-old 304Q/304Q mice compared to WT/WT. Top ten significant terms, their adjusted p-values, and DEG count shown. (D-E) Protein expression of Il20rb, Il33 and Syndig1L in cerebellar lysates of 2- (D+F) and 12-month-old (E+G) mice showed only minimal changes between genotypes and age groups. RNA-seq n = 5, males only; qRT-PCR n = 3, males only; and protein analysis n = 4 both sexes; two-tailed Student's t-test, * or # $p < 0.05$, *** or ### $p = 0.001$, 2m = 2 months and 12m = 12-month-old animals. β-actin was used as loading control

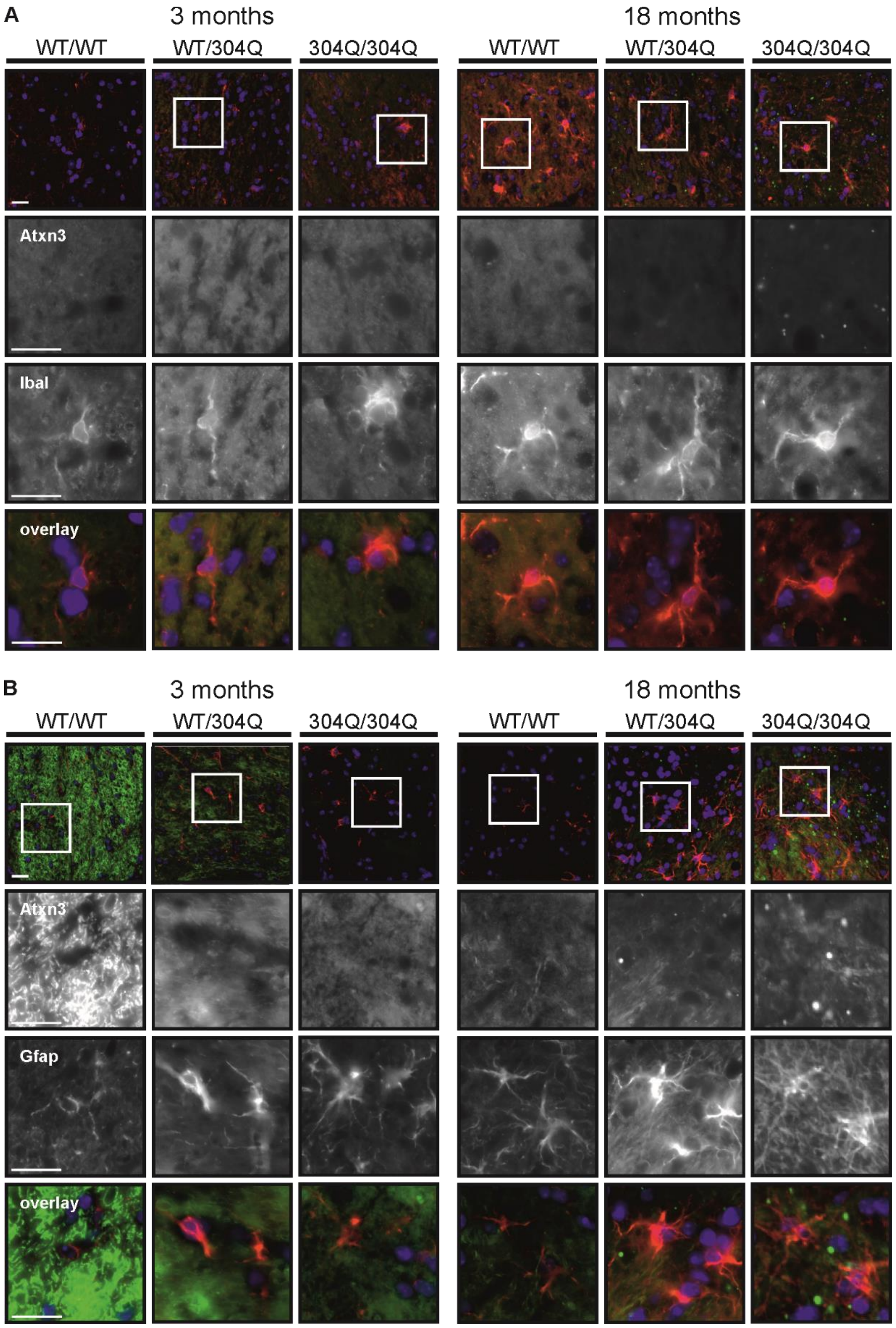

Online Resource SI10 (.tif) Early activation of microglia and astrocytes in WT/304Q and 304Q/304Q mice. (A) Immunofluorescent (IF) staining using IbaI-specific antibody showed early microglia activation in 3-month-old WT/304Q and 304Q/304Q mice, but no differences in 18-month-old mice. (B) IF staining with Gfap-specific antibody revealed both a stronger activation of astrocytes in WT/304Q and 304Q/304Q mice with 3- and 18-months of age. n =1; scale bar = 20 µm

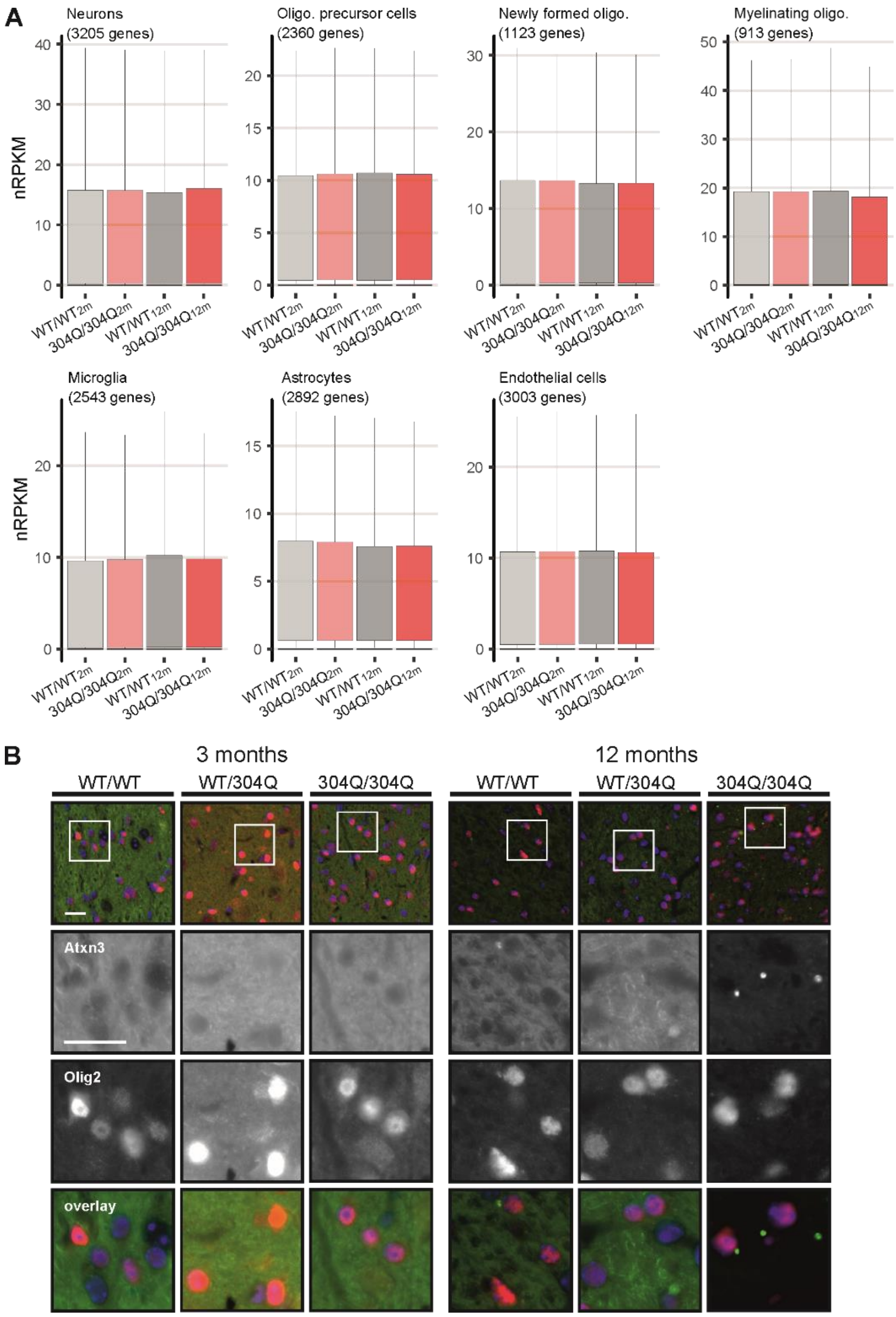

Online Resource SI11 (.tif) Cell type-specific cerebellar gene expression in 2- and 12-month-old WT/WT and 304Q/304Q animals and decreased Olig2 intensity in DCN of KI mice. (A) Boxplots show geometric mean as well as 10th, 25th, 75th, and 90th quantile of nRPKM values for all genes attributed to distinct cell types based on Brain-Seq data from RNASeq analyses in cerebellar lysates [33]. Number of genes per cell type in brackets. No significant compositional changes were observed (Mann-Whitney U test, two-tailed). (B) Immunofluorescent staining of Atxn3 (1H9) and Olig2, showed an increase in the number of Olig2 positive cells in the DCN of 3-months-old mice. Later, with 12 months of age, the numbers of Olig2 positive cells remained unchanged, but the intensity was decreased in WT/304Q and 304Q/304Q mice compared to WT/WT littermates. n = 2; both sexes, scale bar = 20 µm, DCN = deep cerebellar nuclei.
